# Hybrid Multi-Domain ECG Feature Learning with mRMR and CNN–Transformer for Cardiac Disease Classification

**DOI:** 10.3390/bioengineering13091082

**Published:** 2026-09-18

**Authors:** Mohammed Alnusayri, Sara Mumtaz, Bader Aldughayfiq, Hisham Allahem, Nabil Almashfi, Dina Abdulaziz AlHammadi, Ahmad Jalal

**Affiliations:** 1 Department of Computer Science, College of Computer and Information Science, Jouf University, Sakaka 72388, Aljouf, Saudi Arabia; 2 Intelligent Media Center, Islamabad 44000, Pakistan; 232699@students.au.edu.pk; 3 Department of Information Systems, College of Computer Science and Information, Jouf University, Sakaka 72388, Aljouf, Saudi Arabia; bmaldughayfiq@ju.edu.sa (B.A.); allahem@ju.edu.sa (H.A.); 4 Department of Software Engineering, College of Computer Science and Information, Jouf University, Sakaka 72388, Aljouf, Saudi Arabia; nalmashfi@ju.edu.sa; 5 Department of Information Systems, College of Computer and Information Sciences, Princess Nourah Bint Abdulrahman University, P.O. Box 84428, Riyadh 11671, Riyadh, Saudi Arabia; 6 Department of Computer Science, Air University, E-9, Islamabad 44000, Pakistan; 7 Department of Computer Science and Engineering, College of Informatics, Korea University, Seoul 02841, Republic of Korea

**Keywords:** electrocardiogram (ECG), PTB-XL, cardiovascular disease classification, biomedical signal analysis, variational mode decomposition (VMD), multi-domain feature extraction, mRMR feature selection, CNN–Transformer, machine learning, automated diagnosis

## Abstract

Cardiovascular disease diagnosis requires accurate and timely analysis of electrocardiogram (ECG) signals to support reliable clinical decision-making. However, ECG signals are inherently non-stationary, exhibit substantial inter-patient variability, and may share similar morphological patterns across different cardiac disorders, making automated multi-class diagnosis challenging. This study proposes a multi-domain machine learning framework for automated ECG-based cardiac disease classification, integrating signal preprocessing, heartbeat segmentation, Variational Mode Decomposition (VMD), multi-domain feature extraction, minimum Redundancy Maximum Relevance (mRMR) feature selection, and hybrid CNN–Transformer learning. Experiments are conducted on the PTB-XL database using five diagnostic superclasses: NORM, MI, STTC, CD, and HYP. First, a fourth-order Butterworth band-pass filter (0.5–40 Hz) is applied to remove baseline wander and high-frequency noise, followed by adaptive Symlet-8 wavelet denoising with soft thresholding to suppress residual high-frequency fluctuations while preserving the P-wave, QRS complex, and T-wave morphology, after which the signal is z-score normalized. R-peaks are subsequently detected to segment standardized cardiac cycles. VMD is then employed to decompose the heartbeat signals into intrinsic modes, from which the most informative modes are retained using correlation-based mode selection. Temporal, statistical, spectral, and nonlinear features are extracted to capture complementary characteristics of cardiac electrical activity, while mRMR selects the eight most informative features by maximizing feature relevance and minimizing redundancy. The resulting representation is processed through a hybrid CNN–Transformer architecture, in which convolutional layers learn local morphological patterns and Transformer-based attention captures long-range dependencies within the cardiac feature representation. The proposed framework achieves 93.60% accuracy, 93.61% macro precision, 93.60% macro recall, 93.60% macro F1-score, and 98.40% macro specificity across the five diagnostic classes. Confusion-matrix analysis, receiver operating characteristic (ROC) analysis, comparative evaluation, and ablation experiments further demonstrate the discriminative capability and robustness of the proposed approach. These findings indicate that multi-domain biomedical feature learning combined with attention-based deep learning can provide an effective and robust strategy for automated ECG-based cardiac disease classification, highlighting the potential of machine learning for intelligent biomedical signal analysis and computer-aided clinical diagnosis.

## 1. Introduction

Cardiovascular diseases (CVDs) remain among the most significant global public health challenges, encompassing a broad range of disorders, including ischemic heart disease, stroke, hypertensive heart disease, and peripheral artery disease. These conditions affect the cardiovascular system and collectively represent the leading cause of mortality worldwide. According to the World Health Organization, approximately 19.8 million people died from CVDs in 2022 alone [1]. The burden is also substantial at the regional level, with CVDs accounting for more than 42.5% of annual deaths in the WHO European Region, corresponding to approximately 10,000 deaths every day. Furthermore, CVDs are a major contributor to premature mortality among adults younger than 70 years [2,3]. Although a substantial proportion of this burden is associated with modifiable behavioral and environmental risk factors, early and accurate disease detection, risk assessment, and timely clinical management remain essential for reducing complications and mortality. These challenges have increasingly motivated the development of data-driven biomedical technologies capable of supporting clinicians through reliable and automated disease analysis.

Electrocardiography (ECG) is one of the most widely used non-invasive biomedical sensing techniques for assessing cardiac electrical activity. ECG signals provide clinically relevant information for identifying arrhythmias, myocardial ischemia, conduction abnormalities, ventricular hypertrophy, and other cardiovascular disorders. Their low cost, rapid acquisition, and widespread availability make ECG particularly suitable for routine clinical assessment and large-scale biomedical data analysis. However, automated interpretation of ECG remains challenging because cardiac signals are non-stationary, exhibit considerable inter-patient variability, and may contain similar morphological patterns across different disease categories. In addition, manual ECG interpretation requires substantial clinical expertise and can be affected by inter-observer variability, particularly when multiple diagnostic patterns overlap. These challenges highlight the need for machine-learning-based biomedical signal analysis methods that can automatically identify discriminative patterns from ECG data while reducing dependence on manual interpretation.

From a biomedical data-analysis perspective, ECG recordings contain complementary information distributed across temporal, statistical, spectral, and nonlinear domains. However, extracting clinically meaningful representations from these heterogeneous signal characteristics is complicated by baseline wander, muscle artifacts, motion noise, power-line interference, and other acquisition-related disturbances. Conventional signal-processing and feature-engineering approaches may therefore provide limited capability when disease classes exhibit overlapping waveform characteristics. The availability of large-scale, clinically annotated biomedical datasets such as PTB-XL has created new opportunities for developing and systematically evaluating machine-learning approaches for automated ECG classification. In particular, the combination of signal decomposition, multi-domain feature learning, feature selection, and deep neural architectures provides a promising direction for transforming complex ECG measurements into informative representations for disease diagnosis [4].

Although remarkable progress has been made with automated ECG classification, current methods suffer from limitations in extracting complementary signal features, minimizing irrelevant information and learning discriminative representations for multiple cardiac pathologies. While conventional feature-based methods can define key ECG signal properties such as temporal, statistical, spectral or nonlinear properties, they can generate redundant feature spaces, and deep-learning methods predominantly aim at learned representations while neglecting the complementary multi-domain signal information. All of this has inspired an integrated framework to integrate signal decomposition, informative feature characterization, feature selection, and deep representation learning in a single pipeline. To this end, this study proposes a multi-domain framework for automated 5-class ECG classification involving ECG preprocessing and heartbeat segmentation; VMD-based signal decomposition; temporal, statistical, spectral and nonlinear feature extraction; informative and non-redundant feature selection using the mRMR method; and a hybrid CNN–Transformer classifier. In this integration, the framework aims at acquiring a compact and discriminative representation of the ECG and the integration of convolutional feature learning with attention-based modeling for effective classification of NORM, MI, STTC, CD and HYP diagnostic categories.

The main contributions of this study are summarized as follows:

1. A robust ECG preprocessing and heartbeat segmentation pipeline incorporating Butterworth band-pass filtering, R-peak detection, and standardized heartbeat segmentation to improve the quality and consistency of biomedical signal representations.
2. A multi-domain ECG feature-learning framework that integrates Variational Mode Decomposition with temporal, statistical, spectral, and nonlinear descriptors to capture complementary characteristics of cardiac electrical activity.
3. An mRMR-based feature-selection strategy that identifies the most informative ECG characteristics while minimizing feature redundancy, thereby producing a compact and discriminative biomedical representation for downstream classification.
4. A hybrid CNN–Transformer learning architecture that jointly captures local ECG morphological patterns and long-range dependencies for automated five-class cardiac disease classification.
5. Comprehensive experiments are conducted on the PTB-XL biomedical ECG database, covering the five diagnostic superclasses NORM, MI, STTC, CD, and HYP. The effectiveness and robustness of the proposed framework are assessed using classification performance metrics, confusion-matrix analysis, receiver operating characteristic (ROC) analysis, comparative evaluation with existing approaches, and ablation experiments. The resulting findings demonstrate the potential of integrating multi-domain biomedical feature engineering with modern machine-learning and attention-based deep learning for accurate and robust automated ECG disease diagnosis, directly supporting the broader application of machine learning in biomedical data analysis and computer-aided clinical decision support.

## 2. Literature Review

Automated electrocardiogram (ECG) analysis has garnered significant interest as deep learning methods are capable of capturing intricate cardiac patterns and aiding in the identification of cardiovascular abnormalities. Bulbul et al. [5] systematically review and meta-analyzed the deep learning methods used for the diagnosis of cardiovascular diseases using ECGs and observed that there is a growing trend of using convolutional, recurrent, and hybrid networks to interpret ECGs automatically. They also found that the data quality, class imbalance, methodological variability, and generalizability remained significant issues. The results showed that not only the design of the classifiers, but also the use of suitable signal preprocessing, informative representation and robust evaluation procedures were required to achieve reliable classification of ECGs.

Feature-based ECG analysis also remains a valuable way to represent signal characteristics that are useful for diagnosis, in addition to end-to-end deep learning. Tahir et al. combined 1D Haar wavelet representations with statistical features and employed QDA optimized by an artificial neural network, further demonstrating the effectiveness of hybrid feature-learning strategies for physiological and wearable signal analysis [6]. Jalal et al. employed ECG-based GMM feature extraction together with optimization-based classification for physical health monitoring, demonstrating the applicability of feature-driven ECG representations in automated analysis [7]. One-lead ECG signals have been explored by Fira et al. [8] for traditional and sophisticated features such as morphological, temporal, frequency domain, and nonlinear attributes. The study compared various combinations of features for multi-class arrhythmia classification and investigated the benefits of mRMR-based feature selection in the generation of a small informative set of features. Ameen et al. discussed the role of preprocessing, feature extraction, feature selection, and classification methods in ECG and PCG-based cardiovascular disease classification, while reviewing the machine-learning and deep-learning techniques [9]. These studies show that using complementary descriptors that are carefully chosen can significantly decrease the dimensionality of features without discarding diagnostic information [10]. Similarly, Quaid and Jalal employed statistical signal features with a reweighted genetic algorithm for feature optimization and classification, demonstrating the effectiveness of optimized feature representations in sensor-based pattern recognition [11]. Tahir et al. further demonstrated a multi-feature fusion strategy combining statistical, frequency-domain, and wavelet-based representations with optimization techniques for sensor-signal classification [12].

A great deal of research has been conducted on how performance of deep learning is affected by the representation of ECG. Narotamo et al. [13] compared one-dimensional representation of the ECG with two-dimensional image representation with multimodal fusion approach for classification of cardiovascular disease. As a result of their experiments, they demonstrated that the investigated 1D models improved over the corresponding 2D and multimodal models, showing that representational complexity does not necessarily result in better classification. The results highlight the need to retain signal meanings and the necessity of using a representation that is suited to the nature of the ECG data and learning architecture [14].

CNN-based architectures have shown remarkable learning capability on discriminative representations of the ECG. Elyamani et al. [15] used a deep residual 2D convolutional neural network for cardiovascular disease classification and explored the binary, five-class and finer classification settings on PTB-XL. They showed that residual convolutional learning can be applied to the recognition of cardio-vascular diseases from ECG signals. Some ways to proceed that the authors thought to be worthy of future study were data augmentation and further architectural investigation. This work demonstrates that deep CNNs can gain discriminative properties but there are opportunities to enhance representation and processing of ECG information prior to final classification.

Another approach that received attention for the improvement of ECG representation is the heartbeat-centered processing [16] that focuses on the diagnostically relevant parts of the ECG waveform, such as the R-peaks and QRS-complexes. They have taken into account their experiments with raw ECG signal, entropy-based descriptors, extracted QRS complexes and their combinations for two-, five- and 20-class classification problems. The results revealed that the use of entropy-based information and extracted QRS complexes with ECG representation was beneficial over less informative configurations. This paper shows that representative “heartbeat regions” can be extracted by segmentation and different regions of signal information can be integrated to obtain useful signal representations for subsequent deep-learning classification.

Pałczyński et al. [17] also explored the possibility of ECG classification based on a few-shot learning approach using a dataset based on PTB-XL. They used R-wave detection and extracted QRS from 12-lead ECGs, followed by deep-learning-based representation and distance-based classification. Experiments were conducted with two, five, and 20 classes classification. The study showed the feasibility of using QRS-centered representations in a few-shot learning scenario, and highlighted the need to choose diagnostically relevant ECG regions for classification. More recently, Iftikhar et al. introduced an attention-driven Transformer framework integrating multi-domain EEG representations for physiological signal classification, further demonstrating the potential of attention-based learning for complex biosignal analysis. Together, these studies illustrate further advancement of ECG classifiers based on feature engineering, signal representation, heartbeat-focused processing, and deep-learning architectures, but many of the studies are restricted to the individual approaches.

### Research Gap and Contribution of Current Study

Although significant advances have been made in automatic ECG classification, there are still a number of methodological drawbacks in the current literature. The application of deep convolutional learning, handcrafted feature optimization, alternative 1D and 2D signal representations, entropy-based descriptors, R-peak-guided QRS extraction and heartbeat-centered learning have been studied in previous work. Typical methods, however, focus on a different part of the ECG analysis pipeline, instead of ensemble exploiting signal decomposition, complementary multi-domain characterization, redundancy-aware feature optimization and contextual feature-interaction learning in one unified framework. Moreover, having many correlated descriptors might lead to redundant information, and only using end-to-end representations might result in less control over the complementary signal properties fed into the classifier.

To overcome these drawbacks, a novel integrated ECG classification system is proposed, where VMD decomposes the ECG signal into informative oscillatory components, and then extracts complementary temporal, statistical, spectral and nonlinear descriptors. mRMR is then used to narrow down to a small number of features that are highly informative and contain little redundancy. This optimized representation is then fed into a CNN–Transformer architecture, with CNN layers capturing local relationships in the selected-feature representation and multi-head self-attention models capturing contextual relationships between CNN-derived features. The proposed framework includes signal-level decomposition, multi-domain feature characterization, redundancy-aware feature selection, and contextual feature-interaction modeling for five-class cardiovascular disease classification in this way.

## 3. Materials and Methods

### 3.1. System Methodology

The proposed work is an automated deep learning-based ECG signal analysis and heart disease classification with respect to multi-classes using the PTB-XL dataset. The overall methodology is divided into five stages such as ECG signal preprocessing, heartbeat segmentation, feature extraction, feature optimization and disease classification. First, the raw ECG recordings are preprocessed to eliminate baseline wander, power-line interference and high frequency noise while retaining clinically relevant portions of the ECG recording. Accurate localization of R-peaks is then used to segment the enhanced ECG signals into windows centered around the heartbeats. Finally, the segmented heartbeat is decomposed into nearly informative oscillatory modes that preserve the temporal and spectral structure of cardiac activity using Variational Mode Decomposition (VMD). Time–frequency, nonlinear dynamic, recurrence, and morphological information are then extracted from the decomposed ECG signals using multiple complementary feature extraction techniques. The extracted features are optimized and then fed to the hybrid deep learning-based classifier for ultimate heart disease prediction. This is a multi-stage approach that allows us to represent the features effectively and increases the accuracy of classification, whilst at the same time reducing the time needed for computing.

The proposed framework was assessed on a large publicly available dataset of clinical ECGs, the PTB-XL electrocardiography database, which includes 12-lead ECG recordings from 18,885 patients, and 21,837 12-lead ECG recordings [18]. They are 10 s each and can be sampled at 100 and 500 Hz. To avoid losing ECG morphology during pre-processing and heartbeat segmentation the 500 Hz recordings were selected for this study. The PTB-XL database contains expert-annotated diagnostic information, which was used to define five clinically relevant diagnostic superclasses: Normal ECG (NORM), Myocardial Infarction (MI), ST/T Change (STTC), Conduction Disturbance (CD), and Hypertrophy (HYP). These five categories were used for the multi-class ECG classification task.

**Figure 1:**
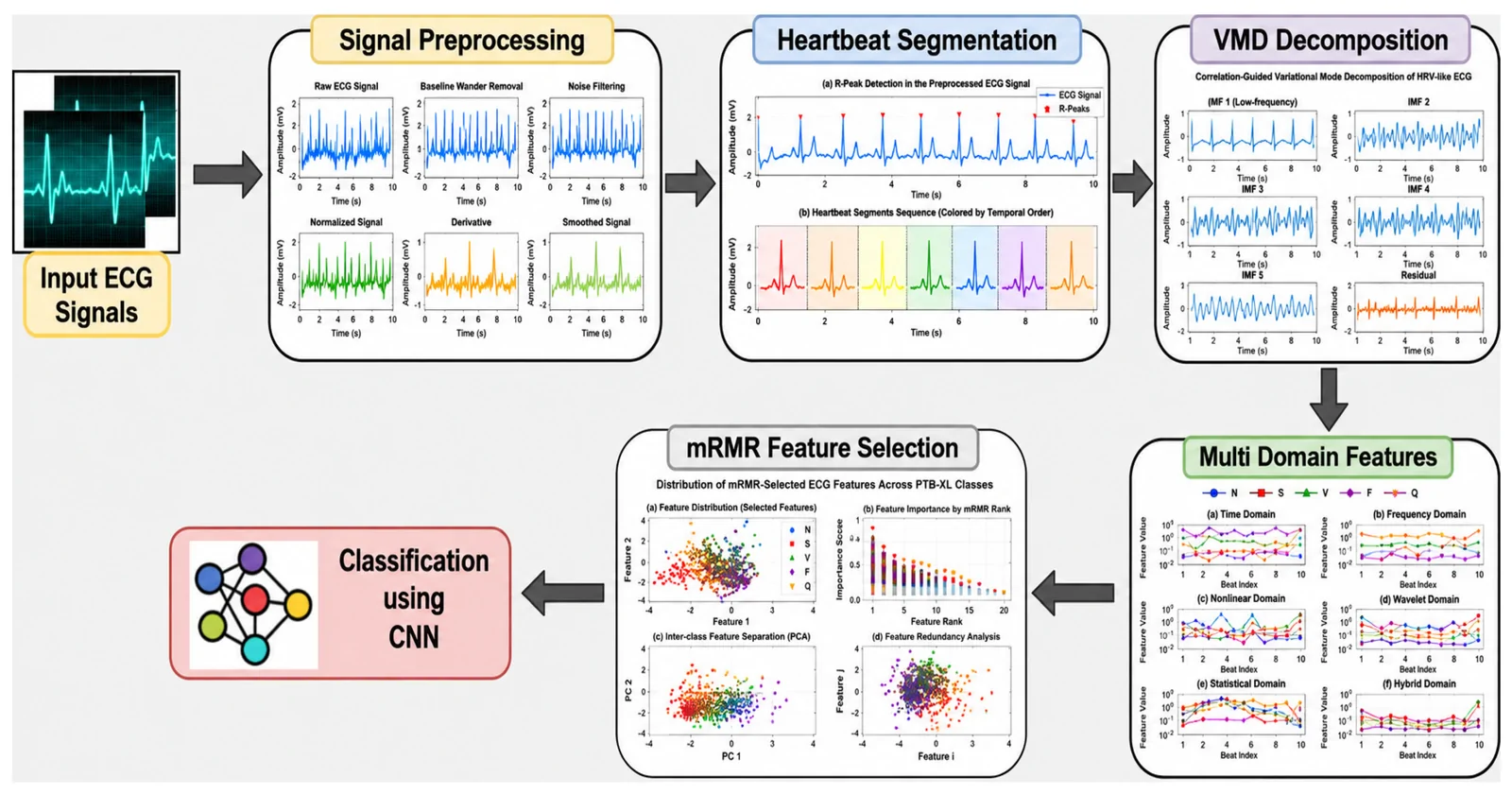
Proposed multi-domain ECG disease classification framework.

The patient-wise partitioning provided with PTB-XL was adhered to in order to guarantee reliable evaluation of the models and prevent patient-level data leakage. The folds 1–8 were used for the training set, fold 9 was used for the validation set and fold 10 was used for the independent test set. In the 5-class classification task, the recordings were re-taken if they contained a clear target diagnostic superclass and other recordings were rejected for potentially ambiguous class assignments. Throughout this process, the patient-wise fold assignments were preserved to ensure that ECG recordings and heartbeat segments in the same patient were in the same dataset partition. If balancing of classes was required, it was applied only to the training data set, but not to the validation and test data sets. Patient-wise partition, preprocessing, VMD, and multi-domain feature extraction were done independently within the training, validation and test partitions without exchanging any information between the subsets. Importantly, the mRMR feature selection was trained only on the training partition (folds 1–8) with just features and labels in the training partition. The eight features that were selected and the fixed order were then transferred to the validation (fold 9) and independent test (fold 10) partitions without any changes. The model development and evaluation were not influenced by any validation or test data for feature selection, class balancing or model fitting, hence no information leakage during the process. A summary of the patient-wise dataset partitioning strategy used in this study is provided in Table 1. The proposed overall classification framework for ECG is shown in the following Figure 1.

### 3.2. ECG Signal Preprocessing

ECG recordings captured from the PTB-XL database are often contaminated with different types of noise and artifacts that can degrade the effectiveness of automated heart disease classification systems [19]. The most frequent sources of interference are baseline wander due to patient respiration, power-line interference from electrical equipment, muscle contractions noise and high-frequency noise introduced during signal acquisition. These unwanted components alter the morphology of the P-wave, QRS complex and T-wave, resulting in improper segmentation of the heartbeat and unreliable extraction of features. For these reasons, the need for an effective pre-processing phase becomes very important to enhance the quality of the signals and maintain clinically important cardiac information.

To tackle these challenges, a two-stage preprocessing approach was used. The baseline wander and the high frequency interference were suppressed by using a fourth order Butterworth bandpass filter, leaving the diagnostically important frequency components of the ECG signal. Then, filtered ECG signal was processed with adaptive Symlet-8 wavelet denoising which successfully eliminated the remaining high frequency fluctuations and preserved the morphology of the P-wave, QRS complex and T-wave. Lastly, all the signals were normalized using z-score normalization to ensure the consistency in the signal amplitude of each ECG record for a better feature extraction and classification.

#### 3.2.1. Butterworth Bandpass Filtering

The first preprocessing step is to eliminate baseline wander from low-frequency and high-frequency noise, while retaining the diagnostic features of the ECG waveform. The clinically relevant portions of the ECG signal are in the range from 0.5 Hz to 40 Hz, so a fourth-order Butterworth band pass filter (BPF) was used to filter out frequencies outside this band [20]. The Butterworth filter was selected due to its maximally flat frequency response in the passband, which minimizes signal distortion while providing efficient attenuation of unwanted frequencies. The frequency response of a Butterworth bandpass filter is expressed according to [20] as:

(1) $$H_{\left(s\right)}=\frac{1}{\sqrt{1+{\left(\frac{s}{w_{c}}\right)}^{2n}}}$$

where $H_{\left(s\right)}$ represents the transfer function of the Butterworth filter, n denotes the filter order, $w_{c}$ is the cutoff angular frequency, and s represents the complex frequency variable.

#### 3.2.2. Adaptive Symlet-8 Wavelet Denoising

Butterworth bandpass filtering can block baseline wander and out-of-band interference, but small high-frequency variations may remain in the filtered ECG records. To further improve signal quality while retaining clinically relevant waveform characteristics, we used adaptive Symlet-8 wavelet denoising as a second preprocessing step. The Symlet-8 wavelet was chosen due to its nearly symmetric basis functions, which give it a good time–frequency localization and morphology of the P-wave, QRS complex, and T-wave [21]. Multi-level wavelet decomposition was used for each ECG signal, and the residual noise was removed by adaptive soft thresholding of the detail coefficients.

**Figure 2:**
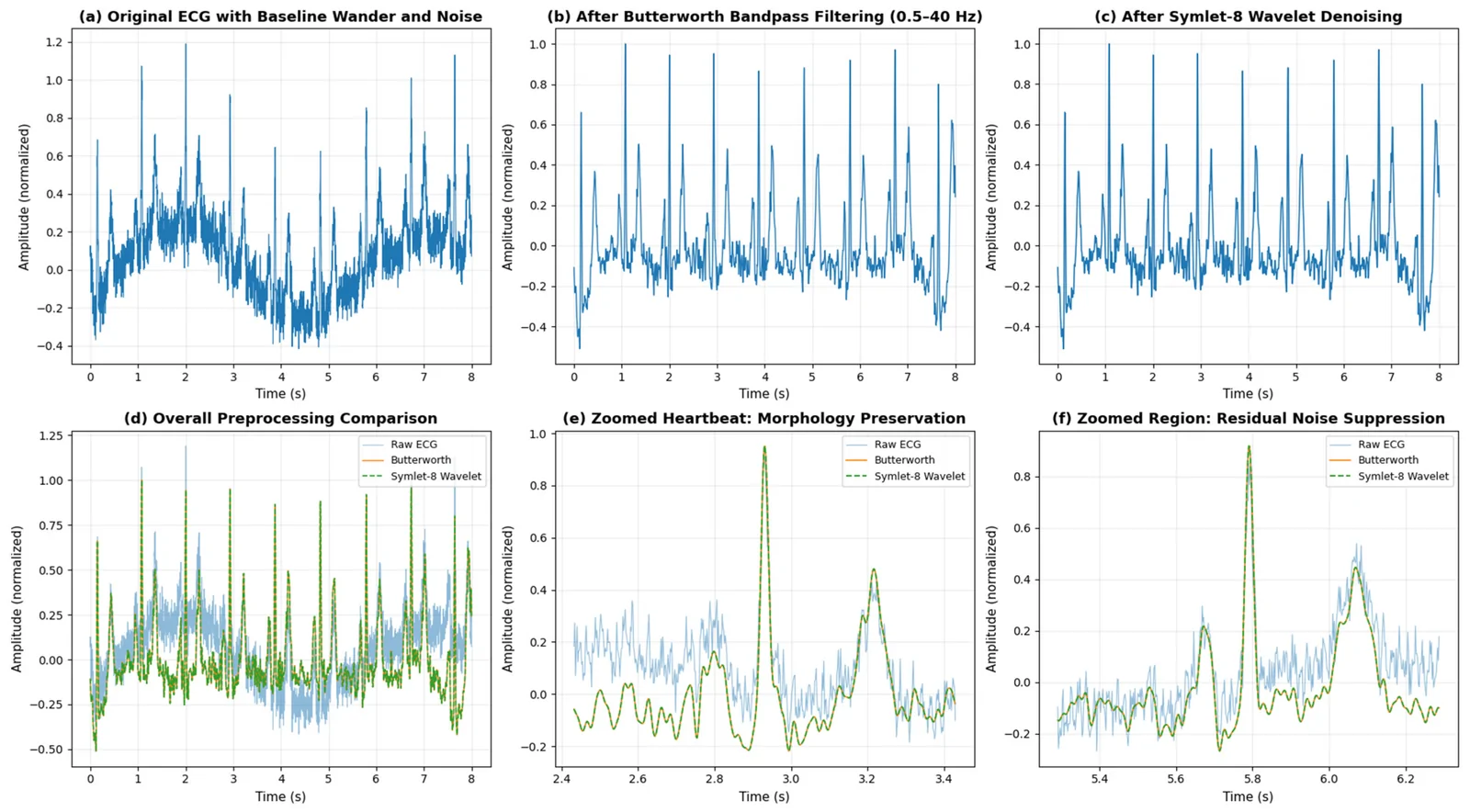
Representative PTB-XL ECG preprocessing results after Butterworth bandpass filtering and adaptive Symlet –8 wavelet denoising.

After smoothing, z score normalization was applied to enhance the consistency of the signals across the different recordings. This normalization makes each ECG record have zero mean and unit variance, which helps to remove amplitude differences between patients. The normalization is expressed according to [22] as:

(2) $$x_{norm}=\frac{x-\mu }{\sigma }$$

where  x represents the preprocessed ECG signal, μ denotes the mean value of the ECG signal, σ represents the standard deviation, and $x_{norm}$ is the normalized ECG signal. This proposed pre-processing pipeline has a good performance in suppressing baseline wander, power-line interference and residual high-frequency noise, while maintaining the clinically relevant morphological features of the ECG signal. The sequential preprocessing steps for a typical PTB-XL ECG recording are shown in Figure 2: original signal, Butterworth bandpass filtering, adaptive Symlet-8 wavelet denoising and zoomed signal comparisons.

### 3.3. Heartbeat Segmentation

The enhanced ECG signal was then segmented into each individual heartbeat-centered signal for reliable cardiac analysis. Heartbeat segmentation breaks the complete ECG signal into individual cardiac cycles by using a fixed-length window around the R-peak and locating the R-peak. The aim of this strategy is to allow the proposed framework to concentrate on the morphological features of individual heartbeats, reducing the computational complexity and maximizing the consistency of the features among various patients [23].

The heartbeat segmentation procedure is divided into two successive steps: automatic R-peak detection and extraction of heartbeat window. The R-peaks associated with the QRS complexes are first detected by using the Pan–Tompkins algorithm. Each R-peak detected is then used as the center of a fixed-length window around it to capture the entire P-wave, QRS complex, and T-wave. The segments obtained are based on the heartbeat and serve as standard inputs to the next step Variational Mode Decomposition (VMD) and multi-domain feature extraction.

#### 3.3.1. R-Peak Detection

The localization of R-peaks is critical since the R-wave has the largest amplitude in the ECG signal and provides a good reference for heartbeat segmentation. The Pan–Tompkins algorithm was used in this study because it is easily implemented and robust in the detection of QRS complexes. After the application of adaptive thresholding and peak se-lection, the algorithm finds local maxima, which represent the depolarization of the ventricles, even if there is residual noise present. The detected R-peak location within the specified search window can be mathematically expressed according to [24] as:

(3) $$R={\arg max}_{t\in W}\;x(t)$$

where R denotes the detected R-peak location, x(t) represents the preprocessed ECG signal, and W indicates the search window around the QRS complex. Only peaks that met the set temporal and amplitude thresholds were used in the subsequent processing, to assure physiologically valid heartbeat intervals.

#### 3.3.2. Heartbeat Window Extraction

**Figure 3:**
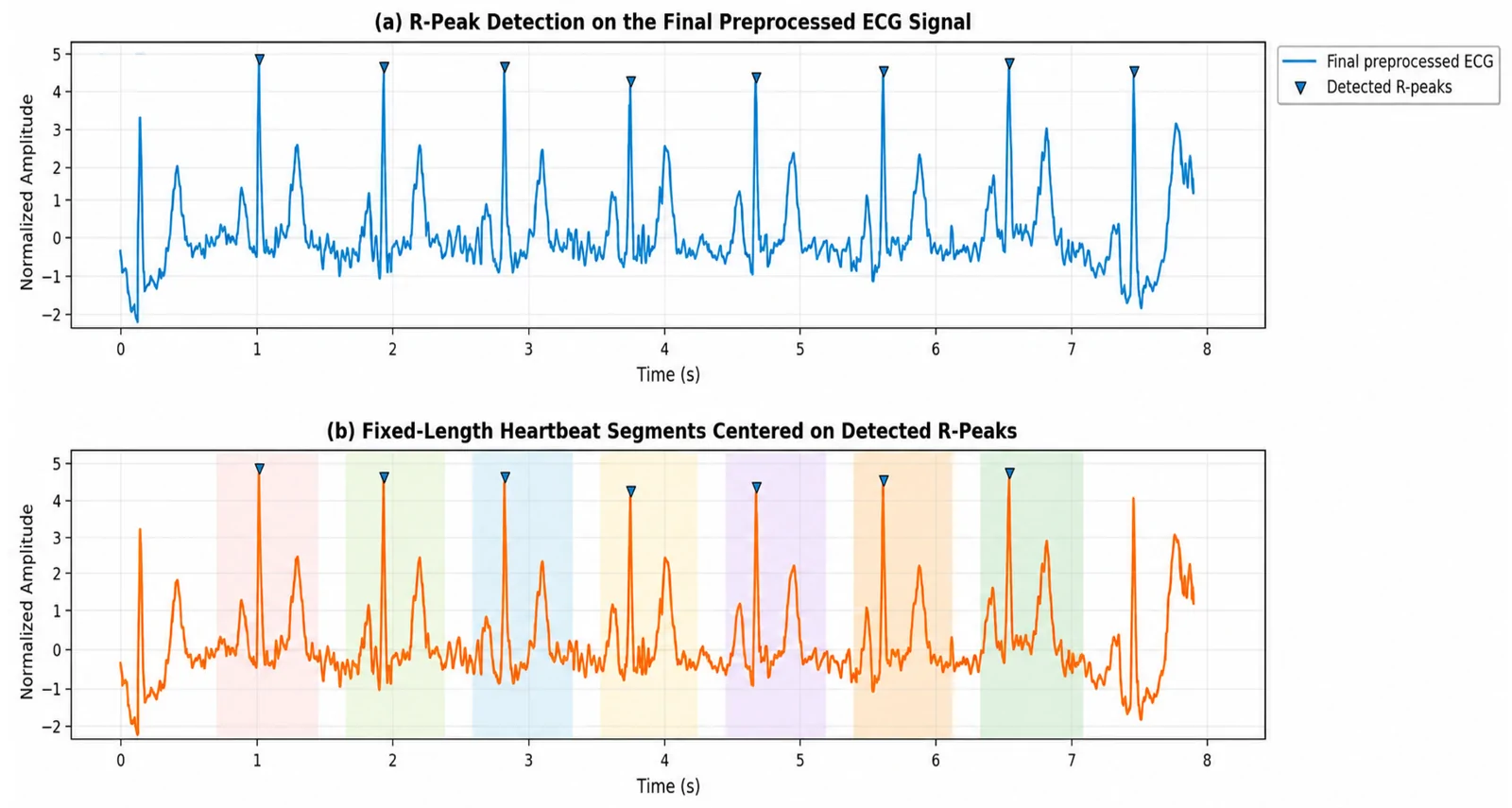
Heartbeat segmentation of a representative PTB-XL ECG signal showing automatic R-peak detection and extraction of heartbeat-centered ECG segments.

The ECG segments corresponding to each heartbeat were extracted after the detection of the R-peak and a fixed length window was selected around each R-peak. This method will allow the whole cardiac cycle to be captured in each segment, including the P-wave, QRS complex and T-wave, which preserves the temporal and morphological information needed for accurate feature extraction. In this study, a symmetric heartbeat window centered at each detected R-peak was used to extract standardized heartbeat segments from all the ECG recordings. Following the R-peak-centered heartbeat segmentation formulation reported in [25], each heartbeat segment was extracted as:

(4) $$S_{i}=[x(R_{i}-N_{b})\;:{x(R}_{i}+N_{a})]$$

where $S_{i}$ represents the extracted heartbeat segment, $R_{i}$ denotes the detected R-peak position, L indicates the predefined window length, and x(.) represents the preprocessed ECG signal. Finally, each individual heartbeat was used as an input to the Variational Mode Decomposition stage for extracting informative oscillatory components for further feature extraction. The heartbeat segmentation process is able to successfully detect the R-peaks and extract the ECG segments with respect to the heartbeat, which retains the entire cardiac morphology but is standardized. Figure 3 displays the segmentation results from a typical ECG signal recorded from the PTB-XL database.

### 3.4. Variational Mode Decomposition (VMD)

**Figure 4:**
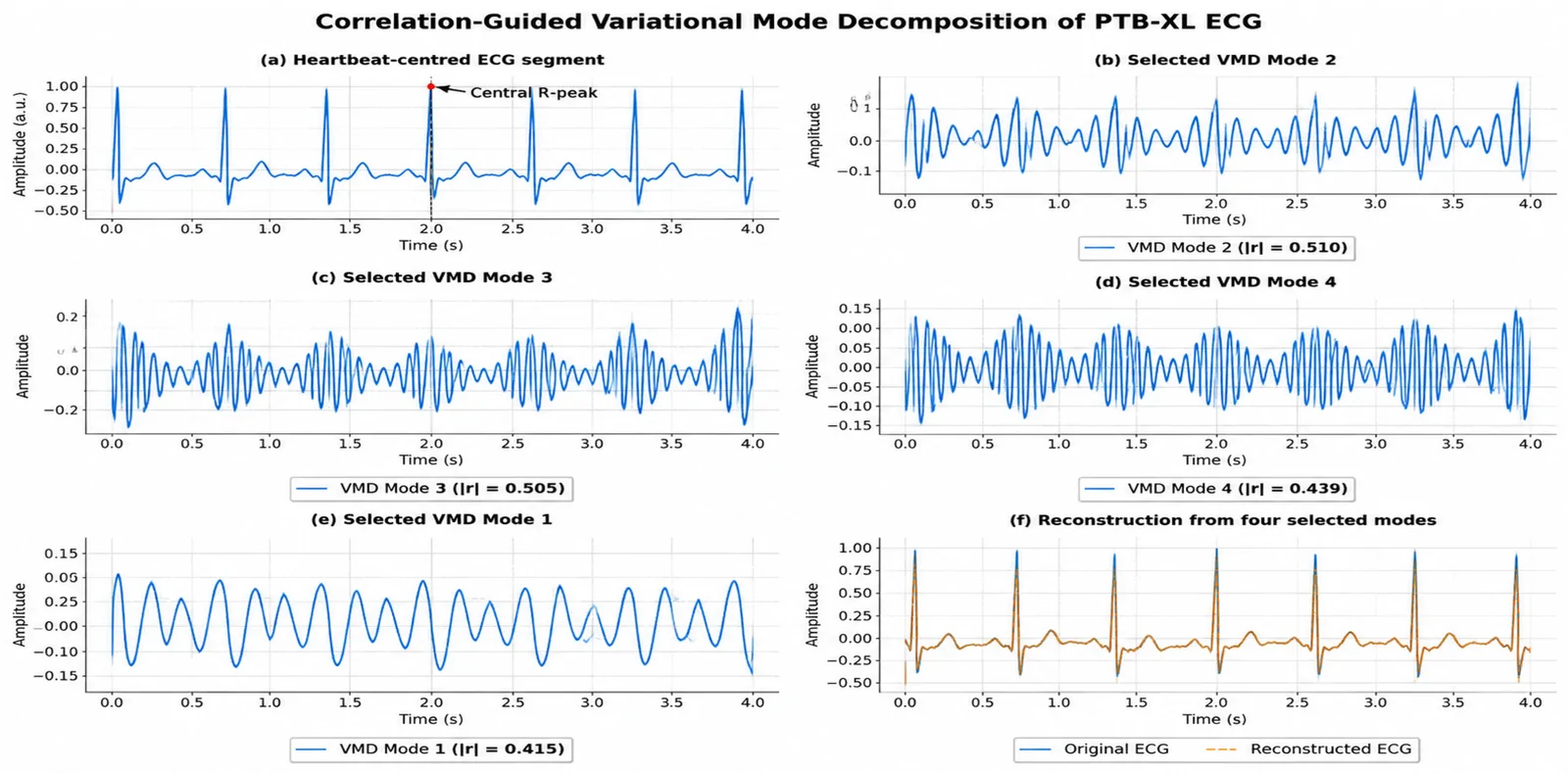
Variational Mode Decomposition of a representative ECG heartbeat showing the original heartbeat, selected VMD modes, and reconstructed signal.

Each segmented heartbeat was then decomposed into a number of band-limited oscillatory modes by using a Variational Mode Decomposition (VMD) technique prior to feature extraction. ECG signals are non-stationary and have multiple frequency bands of various cardiac activities [26]. Direct extraction of features from the original heartbeat might introduce duplicate information and decrease the discriminative ability of the classification model. Hence VMD has been used in this study to decompose ECG signal into several intrinsic modes without compromising the ECG morphological features and mode mixing. In this study, each heartbeat segment has been decomposed into eight variational modes, and the most informative modes have been kept for feature extraction. The constrained variational problem underlying VMD is formulated according to Dragomiretskiy and Zosso [27] as:

(5) $${min}_{\left\{u_{k}\},\{w_{k}\right\}}\left\{\sum_{k=1}^{K}\left\|\partial_{t}\left[\left(\delta \left(t\right)+\frac{j}{\pi t}*\mu k(t)\right)\right]e^{-jw_{k}t}\right\|\begin{array}{c} 2\\ 2 \end{array}\right\}$$

where μk represents the $k^{th}$ decomposed mode, $w_{k}$ denotes its center frequency, K is the total number of modes, and f represents the original heartbeat signal. The similarity of each variational mode and the original heartbeat was determined by the Pearson correlation coefficient after their decomposition. Subsequently, to avoid redundant information, and based on the correlation coefficients of the modes, the four most highly correlated modes were kept and the rest were eliminated for signal reconstruction or feature extraction. To use Pearson correlation as a criterion for signal-preservation, the similarity of each decomposed mode and heartbeats was quantified. Correlation values for the different modes are higher for the modes that contain more of the representative morphological information of the original ECG and lower for the modes that contain less representative oscillatory information or redundant oscillatory information. Importantly, correlation with the original signal was not considered a direct measure of disease relevance. Multi-domain feature extraction and the selection of disease discriminative features using mRMR was then performed. Accordingly, the compact and representative reconstruction of the signal was achieved by selecting the proper modes using the correlation-based VMD method and the features important to the classification of cardiac disease were finally selected in the feature-selection stage. The Pearson correlation coefficient between each decomposed mode and the original ECG signal was calculated according to [28] as:

(6) $$r=\frac{\sum_{i=1}^{N}\left(x_{i}-\bar{x}\right)(y_{i}-\bar{y})}{\sqrt{{\left(x_{i}-\bar{x}\right)}^{2}}\sqrt{{\left(y_{i}-\bar{y}\right)}^{2}}}\;$$

where $x_{i}$ represents the original heartbeat samples, $y_{i}$ denotes the decomposed VMD mode, $\bar{x}$ and $\bar{y}$ are their corresponding mean values, and r is the Pearson correlation coefficient used to identify the most informative oscillatory modes. The segmented ECG heartbeat was used to get the representative VMD results shown in Figure 4.

### 3.5. Multi-Domain Feature Extraction

Selected ECG VMD modes were reconstructed and then converted into a set of complementary handcrafted ECG descriptors that describe diverse physiological aspects of cardiac activity. The proposed framework extracted the temporal, statistical, spectral, and nonlinear features to characterize the ECG signal in different views instead of focusing on one category of features [29]. This full feature representation enhances the accuracy of classification of the subtle variations between normal and abnormal cardiac rhythms and also increases the informativeness of the subsequent feature selection process.

The features in the temporal domain were computed as a description of the amplitude and energy of every sub-epoch of each ECG. The features that were used included the mean amplitude, root mean square (RMS), standard deviation, peak-to-peak amplitude, and the energy of the signal. The temporal descriptors maintain the overall morphology and variations in intensity of the heartbeat, and will be altered by various cardiac disorder.

(7) $$RMS=\sqrt{\frac{1}{N}\sum_{i=1}^{N}x_{i}^{2}}$$

where ${\;x}_{i}$ represents the ECG sample value and N denotes the total number of samples within the sub-epoch.

**Figure 5:**
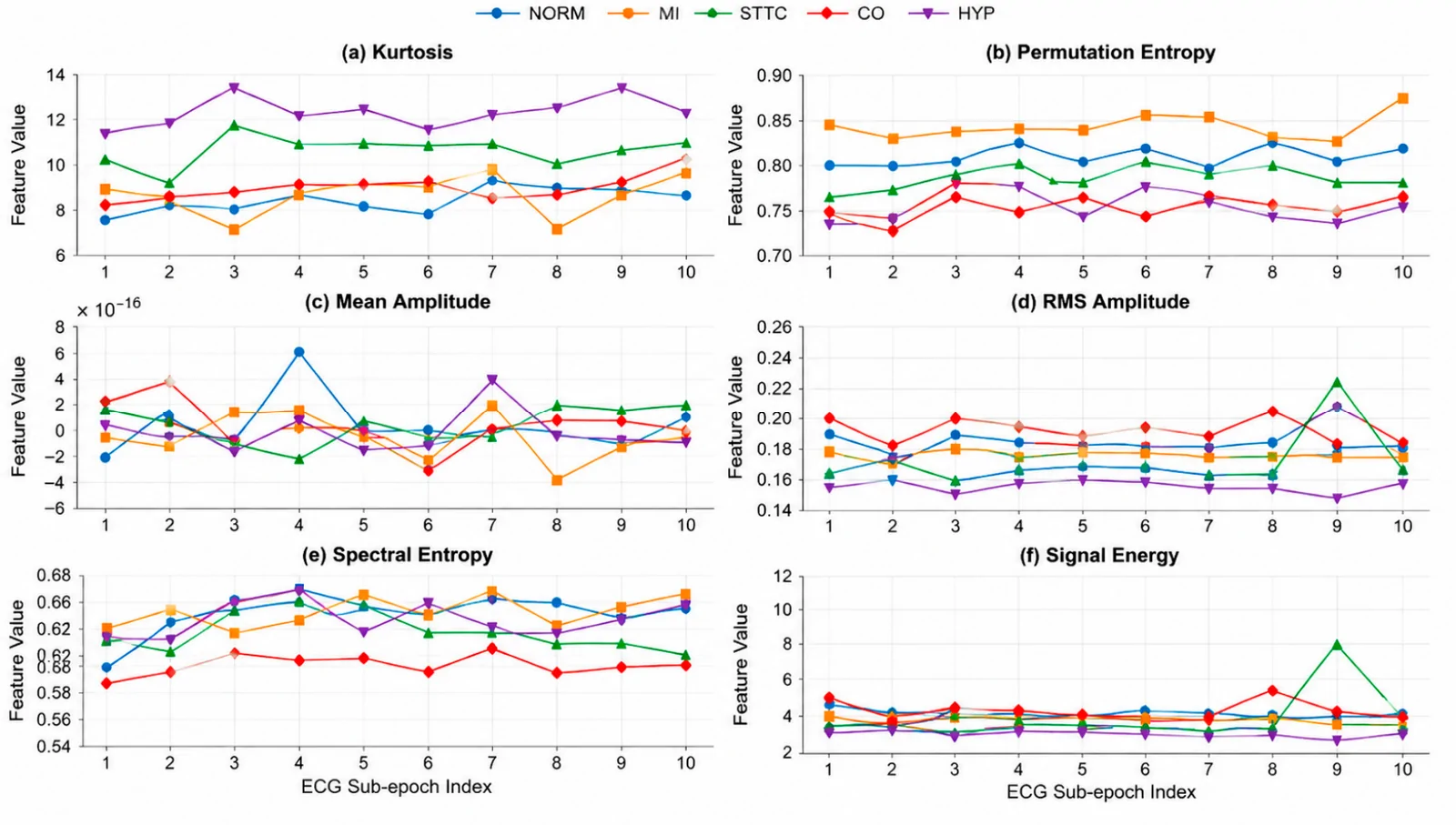
Class-wise variations in representative temporal, statistical, spectral, and nonlinear ECG features extracted from VMD-refined sub-epochs for the five PTB-XL diagnostic classes (NORM, MI, STTC, CD, and HYP).

Then, statistical characteristics were obtained to represent the probability distribution of the ECG amplitude. To measure the asymmetry of the signal, a measure called skewness was used and to measure the sharpness and peakedness of the waveform distribution the measure called kurtosis was used. The following descriptors contain useful information about morphological changes not captured by the amplitude-based measures. Cardiac activity was quantified by computing the dominant frequency, spectral centroid, spectral bandwidth, and spectral entropy. The above features contribute to the discrimination of information as several abnormalities of the cardiovascular system modify the frequency content of the ECG signals. The spectral entropy, which quantifies the distribution of spectral energy within the ECG signal, was calculated according to [30] as:

(8) $$H_{s}=-\sum_{i=1}^{N}P_{i}{log}_{2}(P_{i})$$

where $P_{i}$ represents the normalized spectral power associated with the $i^{th}$ frequency component. Lastly, the complexity and irregularity of ECG dynamics were represented by nonlinear descriptors. The randomness and dynamics of the signal were quantified using permutation entropy and the Hjorth mobility and complexity measures. These non-linear characteristics are used in addition to the temporal and spectral ones, thus providing a more comprehensive representation of cardiac activity. The permutation entropy, which characterizes the nonlinear complexity of the ECG signal based on ordinal patterns, was calculated according to Bandt and Pompe [31] as:

(9) $$PE=-\sum_{j=1}^{M}Pj{log}_{2}(Pj)$$

where Pj denotes the probability of the $\;j^{th}$ ordinal pattern and M is the total number of possible patterns. To study the discriminative behavior of the feature values, those for the five diagnostic classes of ECG (NORM, MI, STTC, CD, HYP) from the VMD-refined ECG sub-epochs were analyzed. The representative feature trajectories for the different diagnostic classes are shown in Figure 5, in which the temporal, statistical, spectral and nonlinear descriptors vary. The combination of these features constitutes the first feature space and is then narrowed down with the proposed feature selection approach for classification.

After the multi-domain feature extraction step, fourteen features, including temporal, statistical, spectral and nonlinear features, were computed for each VMD-refined sub-epoch of the ECG. The selected features were aimed at capturing complementary aspects of the ECG waveform, such as variation in its signal amplitude, morphological asymmetric, frequency distribution, irregularity of the signals and their nonlinear dynamic behavior. The extracted feature set consisted of standard deviation, root mean square amplitude, peak-to-peak amplitude, skewness, kurtosis, signal energy, zero crossing rate, dominant frequency, spectral centroid, spectral bandwidth, spectral entropy, permutation entropy, Hjorth mobility, and Hjorth complexity.

The mean and standard deviation of each extracted feature were calculated for the five diagnostic classes (NORM, MI, STTC, CD and HYP) to describe class-wise variations in the extracted ECG descriptors. Multiple heartbeat segments and VMD refined sub-epochs were not treated as separate patient level samples for statistical inference because they could come from the same patient and were not independent. Therefore descriptive statistics are summarized at the sub-epoch level in Table 2 to describe the distribution of features by the five diagnostic groups observed.

The characteristics extracted in the five diagnostic groups are quite discernible, as illustrated in Table 1. The amplitude descriptors such as standard deviation, RMS amplitude, peak-to-peak amplitude and signal energy show differences in the overall amplitude and variance of the ECG signals. In particular, the CD class shows relatively greater standard deviation, RMS amplitude, peak-to-peak amplitude, and energy values for the signals indicating a greater variation in both amplitude and morphology of abnormalities in conduction.

There are also interesting class-dependent statistical features of the higher order. The STTC and HYP classes have relatively high kurtosis values, which suggests that the signal distributions have a high kurtosis and sharp peaks. Skewness also shows noticeable variation among the diagnostic groups, with the CD group exhibiting a distinctly different distribution compared with the other groups. These differences suggest that there is useful information in the statistical shape of the ECG wave to aid in diagnosis.

The frequency-domain features are based on the differences in the spectral composition of the ECG signals and include the dominant frequency, the spectral centroid and the spectral bandwidth. The dominant-frequency values are relatively high in the STTC and HYP classes, and the spectral centroid and bandwidth are lower in the CD class. Likewise, the difference between the complexity and irregularity of the signals in the classes is quantified using spectral entropy and permutation entropy. In addition to variations in the ECG sub-epochs, there are variations in the dynamics of the behavior seen in the ECG and in the frequency characteristics of the ECG sub-epochs, which are captured by Hjorth mobility and Hjorth complexity, respectively.

The class-wise variations observed suggest that the descriptors extracted possess complementary properties in ECG signals. Some features, however, were very similar and may have redundant information, for example, standard deviation and RMS amplitude. Hence, not all 14 descriptors will lead to a corresponding gain in classification accuracy and the number of features may be raised by introducing all 14 descriptors. The feature set was then further reduced by applying the minimum Redundancy Maximum Relevance (mRMR) feature-selection algorithm in order to obtain a more compact and discriminative feature set.

### 3.6. Minimum Redundancy–Maximum Relevance Feature Selection

The feature pool extracted from the image has valuable temporal, spectral, statistical and nonlinear information, but some of the descriptors may have high correlation, or may provide little diagnostic information to the decision. The addition of such features can lead to computational complexity, introduce multicollinearity and diminish the generalizing power of the classification model. So, the minimum redundancy–maximum relevance approach was used to select a few features with high diagnostic value but without redundant information. mRMR was chosen as the selection method because the multi-domain feature pool is generated from the extracted descriptors and can include redundant information, if not explicitly controlled, which is crucial to reduce the size of the representation. In contrast to NCA, which learns the weights of features based on classification behavior in the neighborhood and ReliefF, which assesses the features based on their diagnostic power to distinguish neighboring instances, mRMR directly balances diagnostic power with inter-feature redundancy using mutual information. This criterion is therefore deemed to be well aligned with the goal of keeping the information complementary to ECG descriptors but not duplicated in the CNN–Transformer classification.

The mRMR method computes the contribution of each candidate feature in two complementary aspects. The first criteria refers to the relevance of an individual feature and the diagnostic class labels. The second is the redundancy from the selected features to the candidate feature. Both measurements use mutual information as it is capable of measuring linear or nonlinear statistical dependencies.

Following the mRMR formulation proposed by Peng et al. [32], the maximum relevance between the selected feature set and the target diagnostic class is defined as:

(10) $$D\left(f_{i}\right)=I(f_{i};C)$$

where $D\left(f_{i}\right)$ denotes the diagnostic relevance of the i-th feature and $I(f_{i};C)$ represents the mutual information between the feature $f_{i}$ and the class variable C. The relevance value is larger the more information it holds for the five diagnostic classes of the ECGs.

The minimum redundancy criterion, which measures the mutual information among the selected features, is expressed as [32]:

(11) $$R\left(f_{i}\right)=\frac{1}{\left|S\right|}\sum_{f_{j}}I(f_{i}:f_{j})$$

where $R\left(f_{i}\right)$ represents the average redundancy of the candidate feature, |S| denotes the number of already selected features, and $I(f_{i}:f_{j})$ represents the mutual information between the candidate feature $f_{i}$ and a previously selected feature $f_{j}$. A high redundancy value means that the feature being tested is carrying information that is provided by the features that have been selected. Finally, the mRMR criterion combines maximum relevance and minimum redundancy by maximizing their difference as [31]:

(12) $${J(f}_{i})=D\left(f_{i}\right)-{R(f}_{i})$$

where ${J(f}_{i})\;$ is the mRMR score assigned to the candidate feature. At every selection step, the feature with the highest value of ${J(f}_{i})$ was added to the optimized feature subset. In the first iteration, the feature with the highest mutual information with diagnostic labels was chosen as there was no feature selected in previous iteration for calculating redundancy with it.

**Figure 6:**
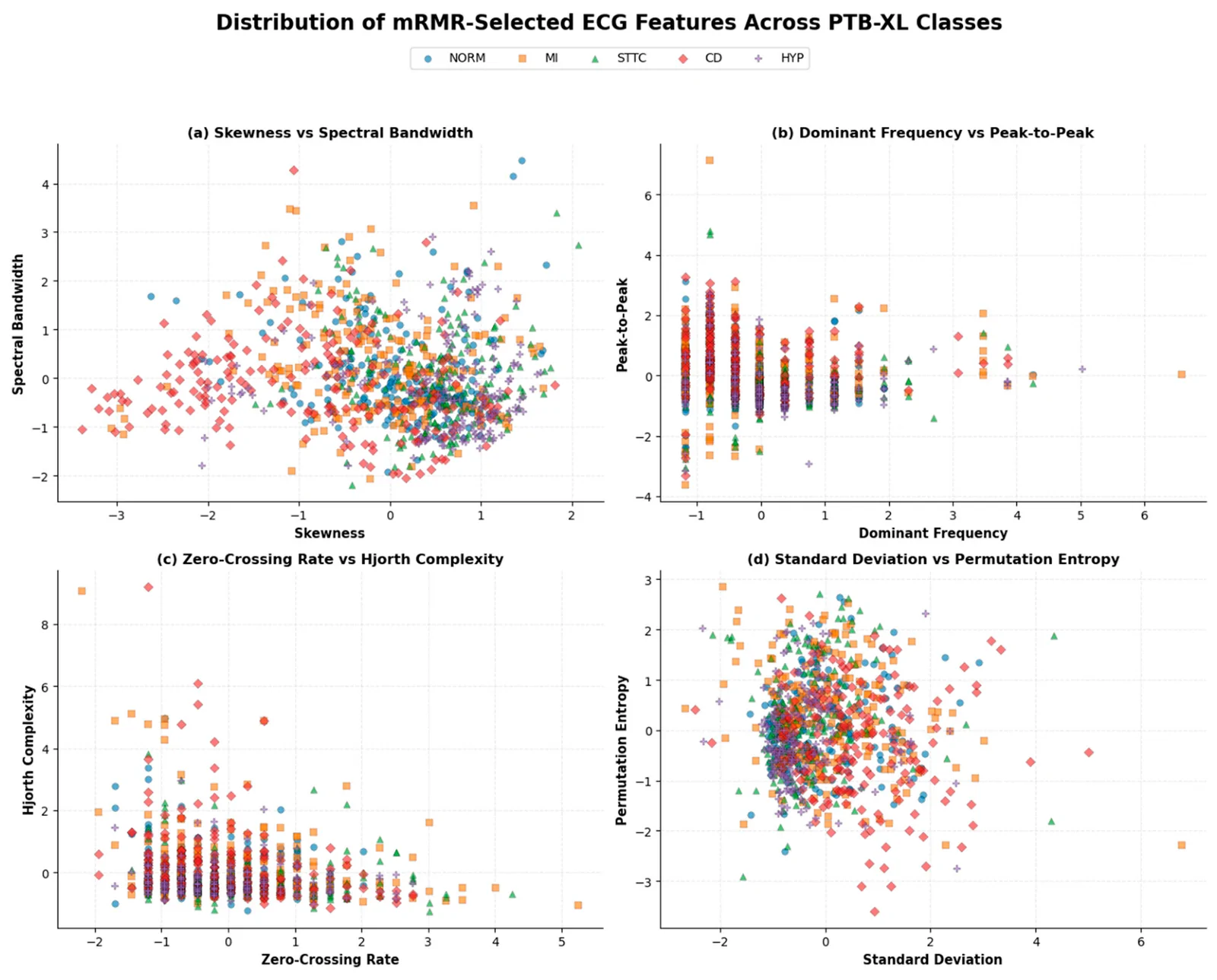
Pairwise distributions of the eight ECG features selected using the minimum redundancy–maximum relevance method across the NORM, MI, STTC, CD, and HYP diagnostic classes.

All 14 extracted descriptors were assessed in an iterative manner with the application of the mRMR criterion. The eight most classifiable features that were still selected for classification were skewness, spectral bandwidth, dominant frequency, peak-to-peak amplitude, zero-crossing rate, Hjorth complexity, standard deviation and permutation entropy. These features are complementary information about the ECG signal: peak-to-peak amplitude and standard deviation are related to amplitude variation and dispersion, skewness is related to the amplitude distribution’s asymmetry, dominant frequency and spectral bandwidth are related to the principal spectral content and the frequency spread, zero-crossing rate is related to the oscillatory variation of the signal, Hjorth complexity is related to the complexity of the ECG signal, and permutation entropy is related to the irregularity of the signal. The selected features are then interpreted as a complementary subset instead of an importance ranking because mRMR takes into account the diagnostic relevance of the features, and they are not redundant among each other. This selected subset was then normalized according to the parameters obtained from the training data, and submitted to the CNN–Transformer classifier. The pair-wise distribution of the mRMR selected ECG feature across the five PTB-XL diagnostic classes are shown in Figure 6.

### 3.7. CNN–Transformer-Based ECG Classification

After feature selection using mRMR, the optimized feature subset of the ECG signals was incorporated into a structured representation and fed into the proposed CNN–Transformer classification network for automated cardiac disease diagnosis. The selected features are the ones which carry the most informative temporal, statistical, spectral, and nonlinear features extracted from each heartbeat, with the least redundant information. This optimized representation allows us to reduce the computational complexity, enhance discrimination between the features, and enable the classifier to learn highly representative patterns related to various cardiac abnormalities [32].

Humanized result: The selected ECG features (after applying mRMR) were arranged in fixed order and fed as an 8 × 1 input to CNN–Transformer classifier. The input representation was first processed by two one-dimensional convolutional blocks. The first block used 64 filters with kernel size 3, the second block used 128 filters with kernel size 3. After each convolutional block, a batch-normalization, ReLU activation and MaxPooling1D were applied. The input to the classifiers is consequently a sequence of length 8, with one channel, with the eight sequence positions corresponding to the fixed order of the features selected by the mRMR for the ECG. MaxPooling1D reduces the dimension of the sequence between the convolutional stages and the final output of the CNN serves as a 128-dimensional representation given to the Transformer encoder. The sequence that is fed into the Transformer is therefore processed as if it were a sequence of positions in the convolutionally transformed selected-feature representation, not a sequence of samples along the time axis of an ECG. The feature sequence was then passed through two 128-dimentional Transformer encoder blocks. All encoders used four heads of self-attention and a dimension of 256 in the feed-forward. The representation of the input was projected to the representations of query, key, and value in multi-head self-attention in each encoder. The attention heads are parallel to learn different relationships between the CNN-derived feature representations and are then concatenated and fed into the 128 dimensional embedding space. The attention output is then added with the encoder input via a residual connection and normalization, then passed through a feed-forward network and a second residual and normalization step. Importantly, the input is compact yet the selected features are complementary temporal, statistical, spectral and nonlinear ECG characteristics [33]. The Transformer is thus used as a feature-interaction modeling unit, with the multi-head self-attention mechanism used to learn the relationship between the feature representations of different parts of the ECG signals, not the relationship between the signals over time [34]. The ablation analysis further validated this by showing that the CNN–Transformer configuration outperformed the CNN-only configuration in terms of classification performance. Global Average Pooling (GAP) was then applied to the Transformer output, reducing the sequence dimension to a fixed length feature vector. This representation was propagated through the fully connected layers of the classification network, which had a dropout rate of 0.5, and then projected onto a five dimensional Softmax output representing the five types of diagnoses. There were 298,885 trainable parameters in the full network [35]. During training, model performance was monitored on the validation set, and the best-performing checkpoint was retained for final evaluation. The detailed architectural and training configurations are summarized in Table 2. The Transformer encoder employs the scaled dot-product attention mechanism introduced by Vaswani et al. [36], which is expressed as:

(13) $$Attention\left(Q,K,V\right)=Softmax\left(\frac{QK^{t}}{\sqrt{d_{k}}}\right)V$$

where Q,K,V denote the query, key, and value matrices, $d_{k}$ represents the key dimension, and the Softmax function computes normalized attention weights for learning feature dependencies. Finally, the learned feature representation is passed through fully connected layers and the output is passed through a Softmax activation function that calculates the probability of each diagnostic category. The Softmax function converts the classifier logits into normalized class probabilities and is expressed according to [37] as:

(14) $$p_{i}=\frac{e^{z_{i}}}{\sum_{j=1}^{C}e^{z_{j}}}$$

where $p_{i}$ denotes the probability of the ith cardiac class, $z_{i}$ is the output logit generated by the classifier, and  C represents the total number of diagnostic classes. To enhance the accuracy of the classification and convergence stability, the cross-entropy loss function in a categorical classification is minimized, and the Adam optimizer is used for network optimization.

**Figure 7:**
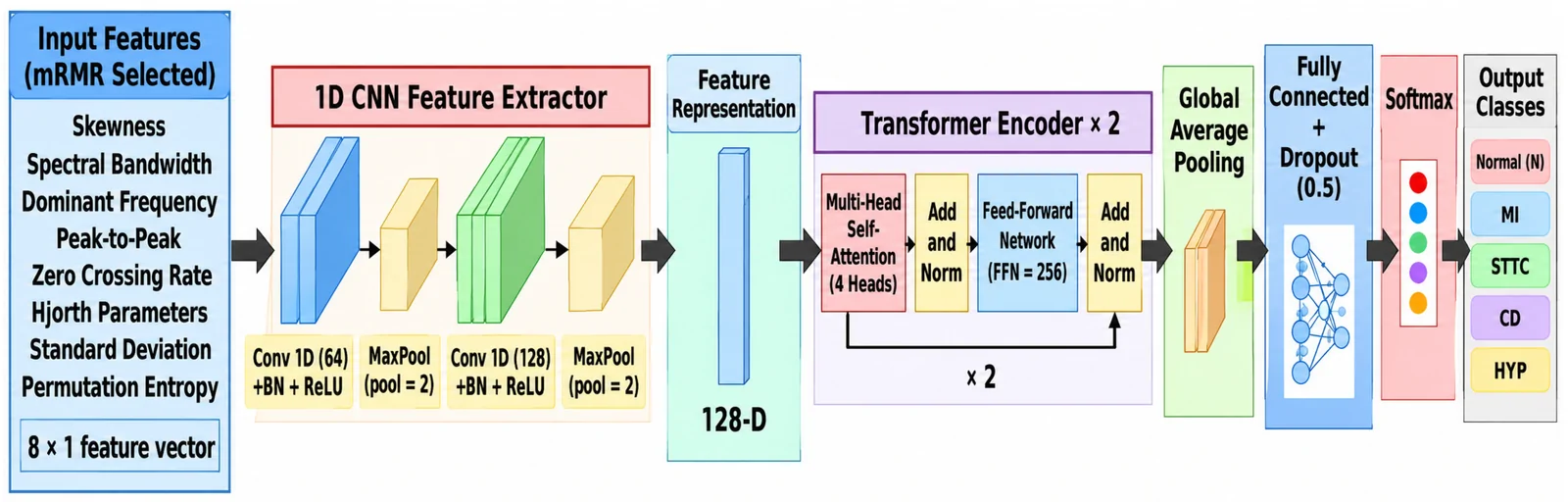
Proposed CNN–Transformer classification framework for automated five-class ECG disease prediction using the optimized mRMR-selected ECG features.

The proposed CNN–Transformer classifier makes the final classification by assigning each ECG segment to one of the five diagnostic categories of PTB-XL such as Normal (NORM), Myocardial Infarction (MI), ST/T Change (STTC), Conduction Disturbance (CD), and Hypertrophy (HYP). The proposed framework provides robust ECG classification performance through the integration of convolutional feature learning and Transformer-based contextual modeling. The proposed CNN–Transformer architecture used for the automated cardiac disease classification of the five classes in PTB-XL is shown in Figure 7.

## 4. Experimental Results and Discussion

The experimental evaluation of the proposed ECG disease classification framework on the PTB-XL dataset is given in this section. First, the experimental setup and the details of implementation are presented and then model training behavior, classification performance, confusion matrix, ROC analysis, visualization of the feature space, and comparison to existing state-of-the-art methods are analyzed. The designed CNN–Transformer framework is thoroughly tested via various quantitative performance metrics.

### 4.1. Experimental Setup

The ECG data set, PTB-XL, was used for all experiments, following the signal preprocessing, heartbeat segmentation, Variational Mode Decomposition (VMD), multi-domain feature extraction and mRMR-based feature selection. This optimized feature representation was then fed into the proposed CNN–Transformer-based five class cardiac disease classification system. In order to provide a reproducible, unbiased evaluation, the official PTB-XL patient-wise data split was followed, using independent training, validation and testing subsets throughout the experimental analysis.

The proposed CNN–Transformer model was programmed in Python (3.13) programming language using deep learning frameworks TensorFlow and Keras. The categorical cross-entropy loss function was used with the Adam optimizer in network optimization. The model convergence was checked by applying the validation data and the model that performed best was used for the final evaluation. The quantitative assessment of the classification performance was done by calculating the Accuracy, Precision, Recall, F1-score, Specificity, Receiver Operating Characteristic (ROC), Area Under the Curve (AUC) and confusion matrix analysis. The experimental setup used in this study is summarized in Table 3.

### 4.2. Model Training Performance

The training behavior of the proposed CNN–Transformer model was studied from the training and validation accuracy curve along with the corresponding loss curve. Throughout the learning process, these learning curves offer insights into the convergence characteristics, optimization stability, and generalization ability of the proposed framework.

**Figure 8:**
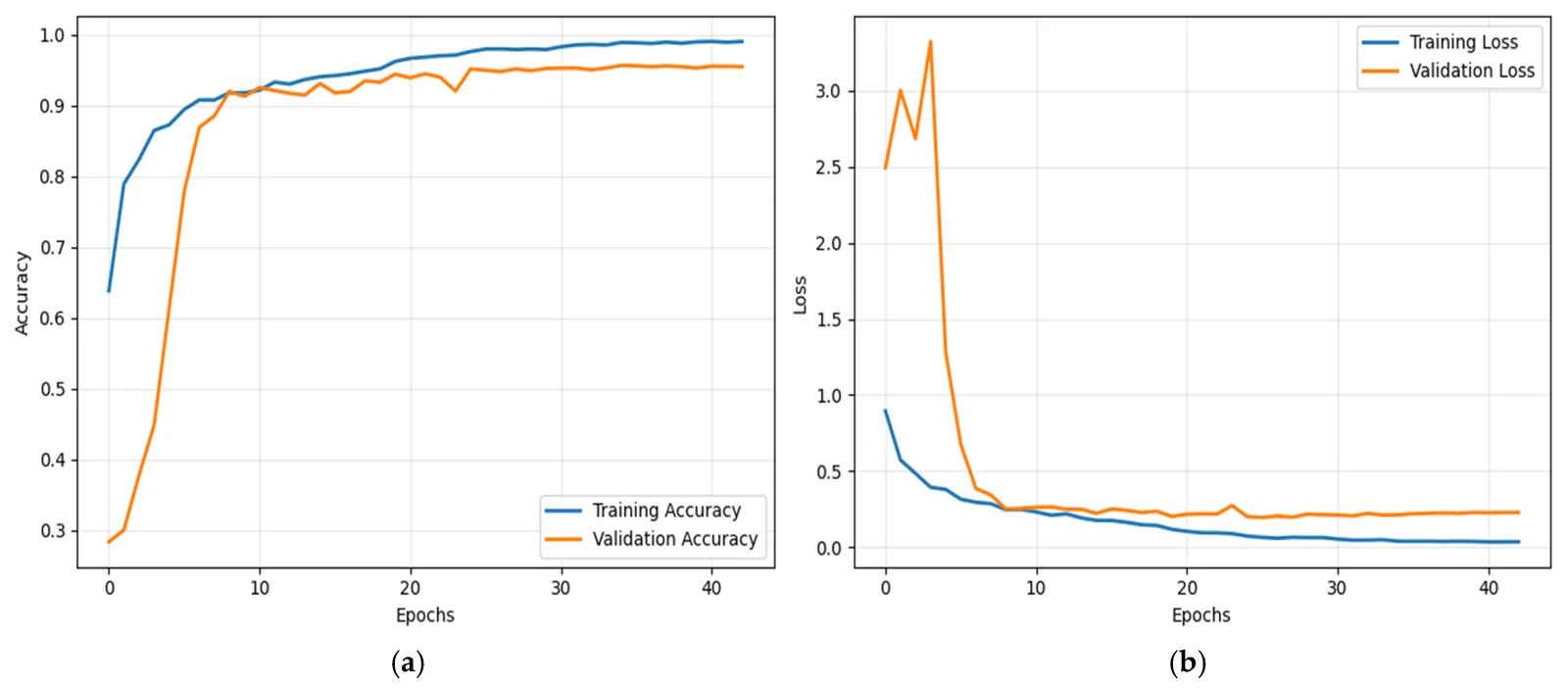
Training performance of the proposed CNN–Transformer model: (**a**) training and validation accuracy curves, and (**b**) training and validation loss curves.

The training accuracy also rapidly rose in the early epochs as shown in Figure 8, which reflects the ability of the CNN layers to learn discriminative local ECG patterns. At the same time, accuracy on the test set showed a steady improvement and eventually caught up with the training accuracy, indicating that learning was stable and not subject to major fluctuations. The slight difference between the two curves suggests that the proposed model was able to predict the ECG samples with good accuracy and reduced the risk of overfitting.

Similarly, the training and validation loss continuously decreased as the number of training epochs increased. The learning curve shows a very fast decrease in the loss in early training, followed by a gradual convergence, indicating an effective optimization of the parameters of the network. The loss curves of both models align again, which further proved that the proposed CNN–Transformer model could learn meaningful ECG feature representations and that there was good convergence of the model in the whole training process. The overall learning curves show the proposed framework to be effective in attaining the convergence of the framework and stable optimization, which also demonstrates good generalization capability, and can be used as a solid basis for multi-class cardiac disease classification. To assess the capabilities of the proposed CNN–Transformer model, the training and validation of the model were carried out with respect to model accuracy and loss in the learning process, which is shown in Figure 8.

### 4.3. Classification Performance Evaluation

The confusion matrix was used to examine the performance of the proposed CNN–Transformer model followed by quantitative analysis with various performance metrics. The confusion matrix shows the classification results in detail and presents a comparison of the predicted labels against the respective ground-truth labels per diagnostic class. It allows for a detailed analysis of both correctly classified and misclassified ECG samples, and is the foundation for deriving quantitative evaluation metrics. The confusion matrix has four basic elements: True Positive (TP), True Negative (TN), False Positive (FP), False Negative (FN). These components detail the prediction result of the classifier, and are used to calculate the remaining performance measures.

- **True Positive (TP):** Number of ECG samples correctly classified as belonging to the target class.
- **True Negative (TN):** Number of ECG samples correctly identified as not belonging to the target class.
- **False Positive (FP):** Number of ECG samples incorrectly assigned to the target class.
- **False Negative (FN):** Number of ECG samples belonging to the target class but incorrectly classified as another class.

**Figure 9:**
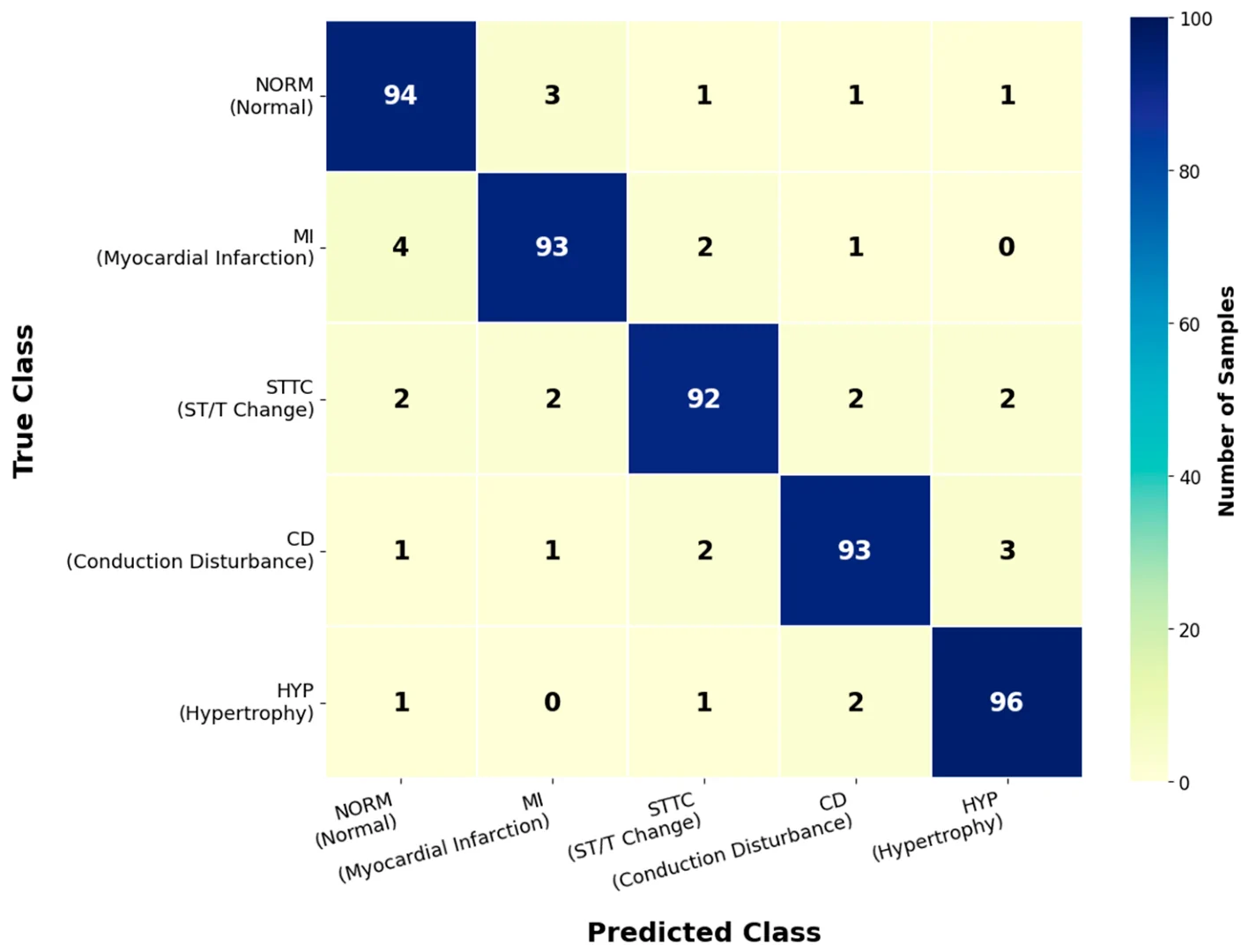
Confusion matrix illustrating the classification performance of the proposed CNN–Transformer model for the five PTB-XL diagnostic classes (NORM, MI, STTC, CD, and HYP).

Figure 9 shows the confusion matrix made with the testing set, which displays the effectiveness of the proposed CNN–Transformer classifier for all five diagnostic categories for PTB-XL.

**Figure 10:**
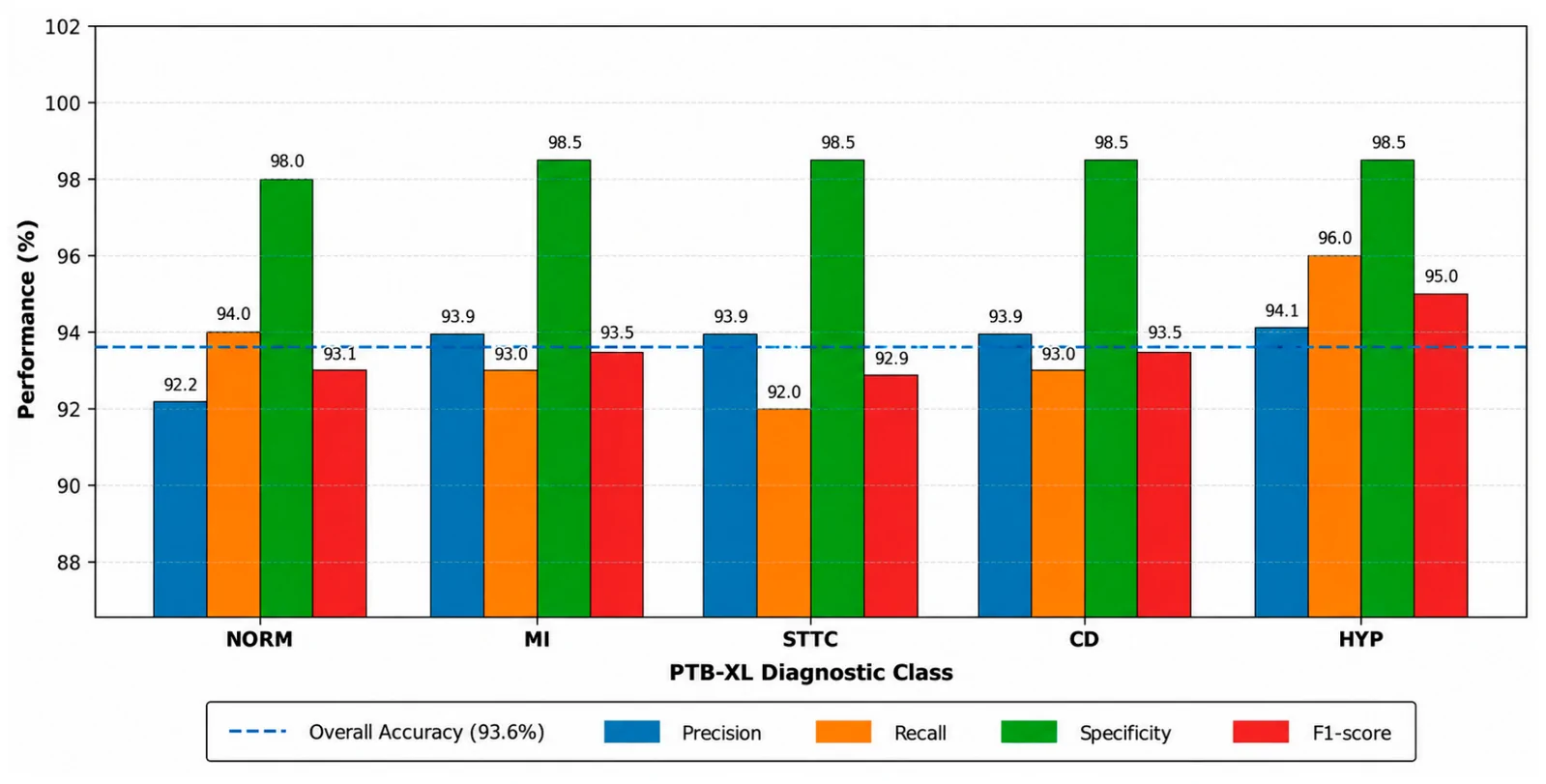
Class-wise quantitative performance of the proposed CNN–Transformer model using Precision, Recall, Specificity, and F1-score across the five PTB-XL diagnostic classes.

The classification accuracy of the proposed CNN–Transformer model for five diagnostic classes of PTB-XL is shown in the confusion matrix presented in Figure 10. The numbers in the confusion matrix are the number of test samples and not percentages. Each row in the evaluation subset sums to 100 samples, as each diagnostic class has 100 samples. The confusion matrix was generated using a balanced evaluation subset of the independent PTB-XL fold-10 test partition, comprising 500 ECG samples, with 100 samples from each of the five diagnostic classes (NORM, MI, STTC, CD, and HYP). These samples belonged exclusively to the independent test partition and were not involved in model training, validation, or feature selection. The balanced class-wise representation was used to provide a clear and directly interpretable visualization of the classification behavior across all five diagnostic categories. Thus, the numerical value in each cell is also numerically equal to the percentage in each of the true classes due to the equal class-wise sample size. So the color bar showing the number of samples is actually showing the absolute number of samples. The concentration of samples on the main diagonal demonstrates that the proposed framework has a high discriminative ability with a large number of ECG records being correctly classified. The classes NORM and HYP had the best correct classification results—94% and 96%, respectively—and the classes MI and CD gave correct classification results of 93% each. The classification accuracy of the STTC class was slightly lower (92%), but still acceptable with few samples being misclassified into the neighboring diagnostic classes. The off-diagonal values are very few in number, which means there is not much confusion between the five cardiac conditions, and indicates that the integration of VMD-based feature extraction, mRMR feature selection and CNN–Transformer classifier was able to learn highly discriminative ECG representations. The confusion matrix further validates the effectiveness and reliability of the proposed approach for multi-class ECG disease classification with an overall classification accuracy of ~93.6% on the PTB-XL dataset.

#### Quantitative Performance Evaluation

After performing the confusion matrix analysis, the quantitative performance of the proposed CNN–Transformer model was assessed via various classification metrics, such as Accuracy, Precision, Recall, Specificity, and F1-score. The overall prediction capability of the classifier has been measured by these evaluation measures, as well as its capability to identify single cardiac disease categories correctly. All the evaluation metrics were directly calculated from the True Positive (TP), True Negative (TN), False Positive (FP) and False Negative (FN) values derived from the confusion matrix.

The overall classification accuracy is obtained as the number of correctly classified ECG samples divided by the total number of ECG samples in the test set and calculated by Equation (15).

(15) $$Accuracy=\frac{TP+TN}{TP+TN+FP+FN}$$

Precision measures the proportion of correctly predicted positive ECG samples among all samples predicted as positive and is defined by Equation (16).

(16) $$Precision=\frac{TP}{TP+FP}$$

Recall evaluates the ability of the proposed classifier to correctly identify actual positive ECG samples and is expressed using Equation (17).

(17) $$Recall=\frac{TP}{TP+FN}$$

Specificity measures the capability of the classifier to correctly recognize negative ECG samples and is calculated using Equation (18).

(18) $$Specificity=\frac{TN}{TN+FP}$$

The F1-score represents the harmonic mean of Precision and Recall and provides a balanced evaluation of the classifier, particularly for multi-class classification problems. It is calculated using Equation (19).

(19) $$F1=\frac{2\times Precision\times Recall}{Precision+Recall}$$

Table 4 provides a summary of the quantitative performance results of the CNN–Transformer model. The proposed framework achieved the overall classification accuracy of 93.60%, which shows its effectiveness in differentiating the five diagnostic classes of the PTB-XL. Moreover, the Precision, Recall, F1-score And Specificity were obtained by the classifier as 93.61%, 93.60%, 93.60% and 98.40% respectively while the macro-averaged value showed 93.60%, 93.60%, 93.60% and 98.40% respectively, thereby indicating stable and reliable performance across all cardiac diseases categories. The consistently excellent evaluation metrics further validate the VMD-based feature extraction method, the mRMR feature selection method, and the CNN–Transformer-based classifier for learning powerful discriminative ECG representations and robust multi-class cardiac disease classification. The quantitative performance of the proposed CNN–Transformer model is shown in Figure 10 for all five diagnostic classes of PTB-XL.

As shown in Figure 10, the proposed CNN–Transformer model had high classification performance for all of the five diagnostic classes of PTB-XL. The HYP class had the highest Recall (96.0%) and F1 (95.0%), which showed better recognition ability for hypertrophy cases. The NORM, MI, STTC, and CD classes exhibited similar performances, ranging from Precision of 92.2% to 94.1%, Recall of 92.0% to 94.0%, and F1-scores of >92.9%. Also, no matter the class, the Specificity remained high (98.0–98.5%), which indicates that the model was very successful in predicting the negative samples correctly and had low false-positive rate. The dashed horizontal line is the overall classification accuracy of 93.6%, which shows that the proposed framework performs well and consistently in all diagnostic categories.

### 4.4. Receiver Operating Characteristic (ROC) Analysis

To further evaluate the discriminative power of the proposed CNN–Transformer framework in the cardiac disease classification problem with five classes, Receiver Operating Characteristic (ROC) analysis was conducted. The ROC curve is not like the confusion matrix, which plots the performance of classification against a single decision threshold; instead, the ROC curve is a plot of the True Positive Rate (TPR) and the False Positive Rate (FPR) as the decision threshold varies. This analysis will present a detailed assessment of the classifier’s sensitivity and specificity and measure the classifier’s discriminatory power between various categories of cardiac disease.

**Figure 11:**
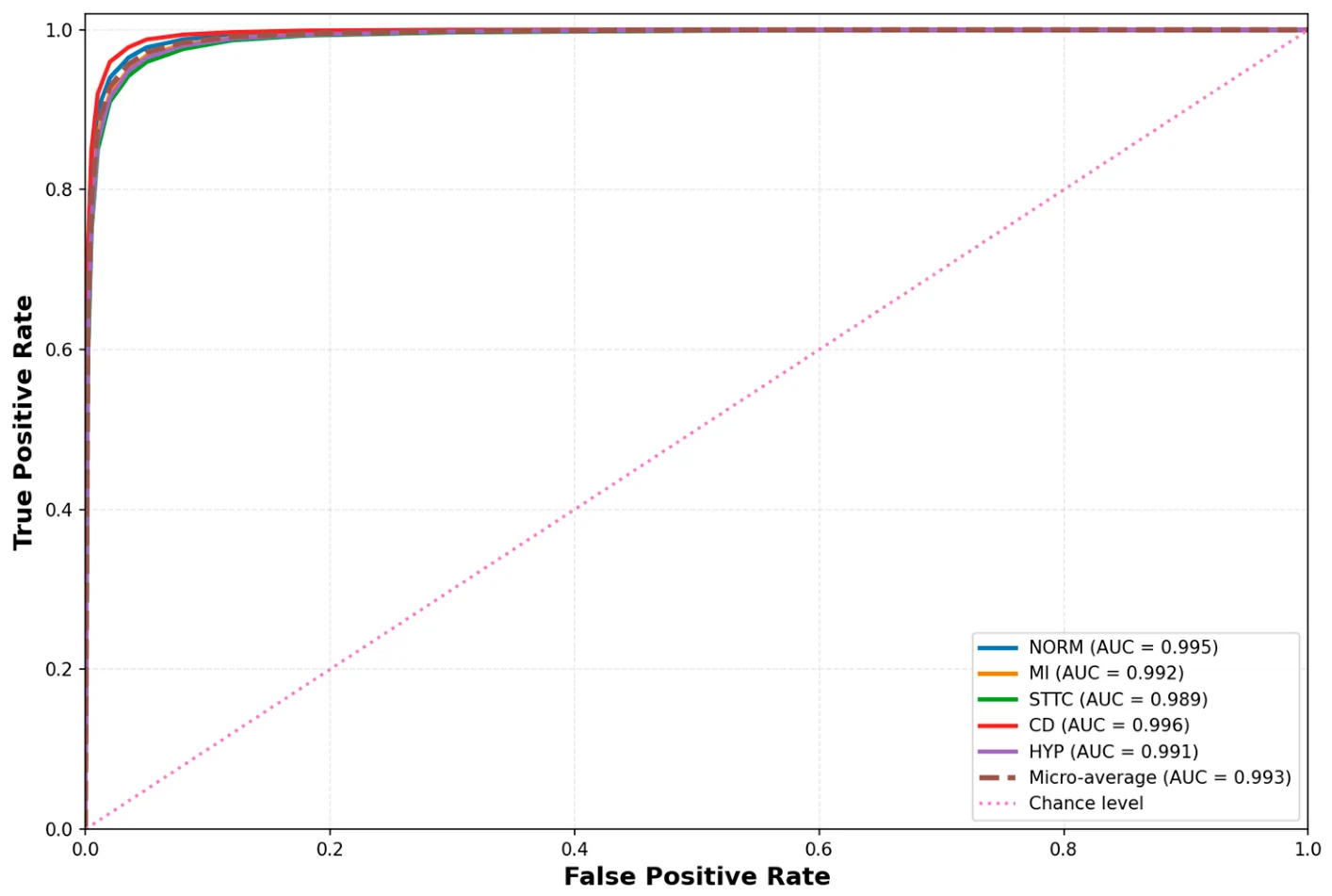
Multiclass Receiver Operating Characteristic (ROC) curves and corresponding Area Under the Curve (AUC) values of the proposed CNN–Transformer model.

Moreover, all ROC curves gather around the top-left corner of the ROC space and are far from the reference line, further demonstrating the good generalization power of the proposed model. Furthermore, the high AUC scores across all the diagnostic classes further confirm the usefulness of using multi-domain ECG feature extraction, mRMR-based feature selection, and the CNN–Transformer model for a robust automated cardiac disease diagnosis. The Receiver Operating Characteristic (ROC) curve plots are shown in Figure 11 for the five PTB-XL diagnostic classes for the proposed CNN–Transformer model.

### 4.5. Feature Space Visualization Using t-SNE

**Figure 12:**
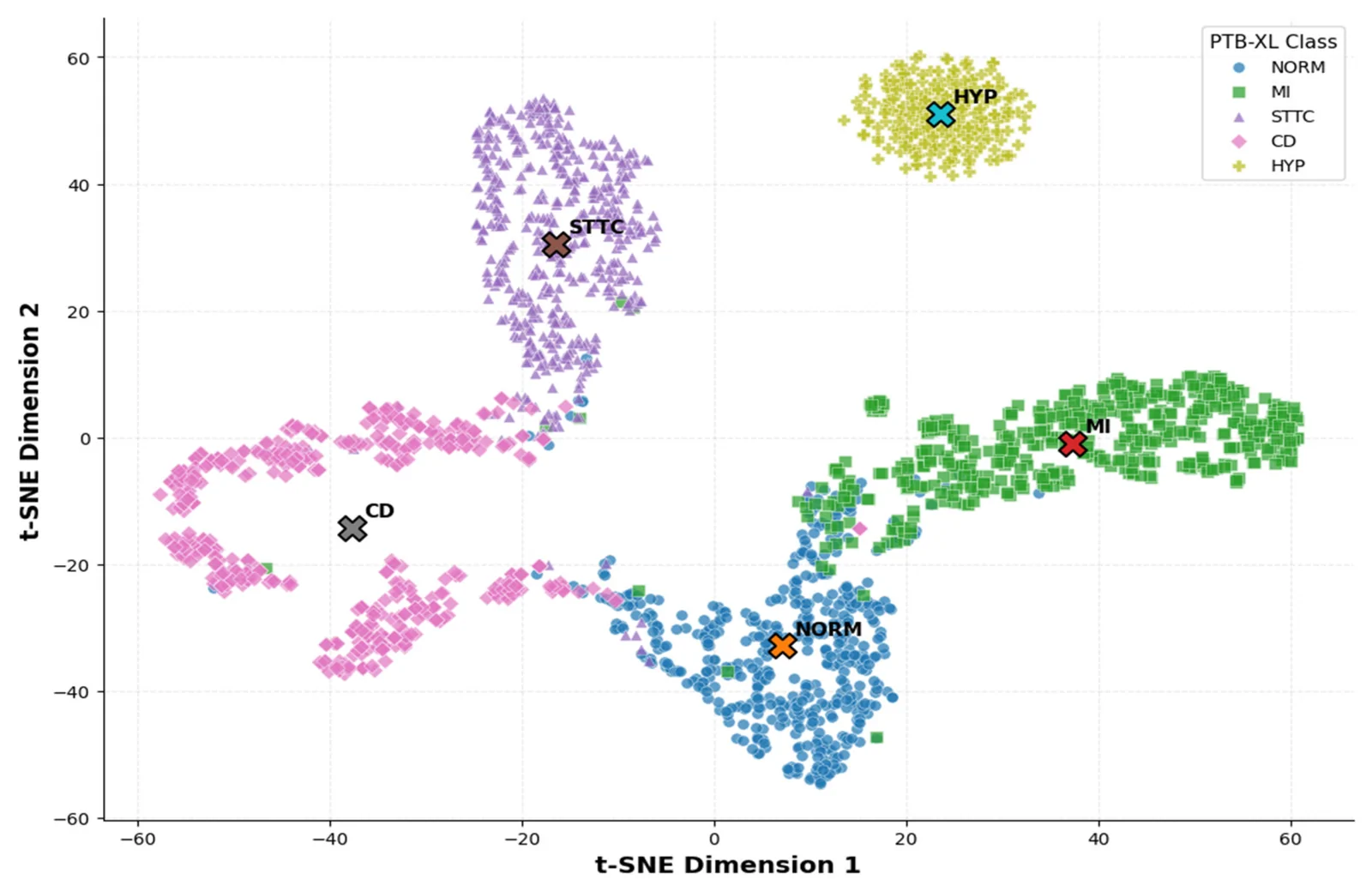
t-SNE visualization of the learned feature representations for five-class ECG disease classification on the PTB-XL dataset.

The feature representations learned by the proposed CNN–Transformer model for the five PTB-XL diagnostic classes are shown in the t-SNE visualization in Figure 12. The clusters in the reduced two-dimensional feature space show the different categories of cardiac diseases. One can see that the learned representations are spread out and class labels are not overlapping except for very small margins between neighboring classes. This means that the suggested framework performs well in learning highly discriminative feature representations of Normal (NORM), Myocardial Infarction (MI), ST/T Change (STTC), Conduction Disturbance (CD), and Hypertrophy (HYP). The results in terms of compact intra-class distribution and clear inter-class separation show the effectiveness of the combination of multi-domain ECG features, mRMR feature selection, and CNN–Transformer classifier. The results again confirm the good classification performance in the confusion matrix and in the ROC analysis.

### 4.6. Comparative Performance Analysis

The performance of the proposed CNN–Transformer approach was then compared with the performance of several existing deep learning approaches reported for cardiac disease classification using ECG images, to further validate its effectiveness. Commonly used evaluation measures such as classification accuracy, Precision, Recall, F1-score, and Area Under the ROC Curve (AUC) were used for the comparison. The purpose of this comparison is to highlight the enhancements realized by using the multi-domain feature extraction and mRMR feature selection in a unified classification framework with CNN–Transformer classifier.

The proposed model exhibited an overall classification accuracy of 93.60%, macro Precision of 93.61%, macro Recall of 93.60%, macro F1-score of 93.60% and micro-average AUC of 0.993 as summarized in Table 5. The proposed framework can achieve competitive classification performance with balanced recognition performance across all five diagnostic classes of PTB-XL compared to the previously reported methods. Convolutional feature extraction along with the transformer-based attention mechanism allows the model to learn the local morphological features and long-range temporal relationships in ECG signals, which enhances its ability for diagnostic discrimination.

In addition, the use of mRMR feature selection limited redundant information prior to the classification and directed the CNN–Transformer model to only concentrate on the most informative ECG characteristics. This helps to improve the generalization results and the stability of the classification in various categories of cardiac diseases. The achieved results prove the proposed framework is capable of achieving a good balance between classification accuracy, robustness, and computational efficiency, which is suitable for an automated cardiac disease diagnosis based on an ECG. Table 5 presents the comparative performance of the proposed CNN–Transformer framework against recently reported ECG classification methods.

Table 5 shows a contextual comparison of the PTB-XL-based ECG classification approaches that have been previously reported with respect to the classification task, data-splitting strategy and evaluation unit. All studies are performed with the PTB-XL but the reported performance numbers are not strictly comparable due to variations in class formulation, patient/record selection, data partitioning, and evaluation metrics. In particular, the diagnostic task in some previous studies is modeled as multi-label classification, while the present study uses mutually exclusive five-class classification using patient-disjoint training, validation, and test partitions. Thus, the findings presented in Table 5 should be viewed as contextual rather than head-to-head benchmarking.

### 4.7. Ablation Study

An ablation study was performed to see how much each major component of the proposed framework was contributing to the system, while all other network configurations were kept constant and only certain components were gradually removed from it. This experiment aimed to compare the CNN backbone, Transformer encoder and mRMR feature selection on the overall classification performance.

All ablation configurations were evaluated using the complete independent PTB-XL fold-10 test partition under the same patient-disjoint data-splitting protocol. The balanced 500-sample subset was used only for confusion-matrix visualization and was not used to calculate the ablation accuracies reported in the table. Each ablation configuration was evaluated once, and no averaging across repeated experimental runs was performed. The results from the ablation experiment are presented in Table 6.

The proposed framework component-wise ablation analysis is presented in Table 6. The CNN baseline was 88.41% accurate, and 91.82% accurate with the Transformer encoder. The CNN baseline was also improved to 90.74% (2.33% improvement on the baseline) by incorporating mRMR. The accuracy after the removal of VMD was 87.69%, and when temporal and statistical features were excluded, the accuracy was 90.34% and 92.62%, respectively. The accuracy of the complete CNN–Transformer framework with mRMR is the highest (93.60%) suggesting the complementary contribution of the evaluated components to the overall classification performance.

### 4.8. Discussion

The experimental findings prove the proposed CNN–Transformer model is reliable and robust to classify five classes of ECG diseases on the PTB-XL dataset. This multi-domain ECG feature extraction, together with mRMR feature selection, and using CNN–Transformer for classification was able to learn both the local morphological features and the long-range temporal dependence, achieving an overall classification accuracy of 93.60%.

The results of the confusion matrix and quantitative performance indicators show that the proposed framework performed well in terms of classification accuracy for all the diagnostic classes with high values of Precision, Recall, and F1-score and high Specificity. Furthermore, the ROC analysis showed high AUC values for all the classes, which reflected the good generalization ability of the proposed model. The ablation study also confirmed that every module positively aided the performance of the model, and the combination of the CNN backbone, the Transformer module, and mRMR feature selection achieved the highest classification accuracy.

The proposed framework showed promising performance but it was tested just on the PTB-XL dataset. The model will be further tested on other public ECG databases, optimized for real-time clinical use and integrated with explainable artificial intelligence (XAI) methods to enhance the interpretability and clinical value of automated ECG diagnostic systems.

## 5. Conclusions

This study presents a machine-learning-based approach for automatic five-class ECG disease classification using the PTB-XL biomedical database. The proposed methodology integrates ECG signal preprocessing, multi-domain feature extraction, mRMR-based feature selection, and a hybrid CNN–Transformer network to classify five cardiac diagnostic superclasses, namely Normal (NORM), Myocardial Infarction (MI), ST/T Change (STTC), Conduction Disturbance (CD), and Hypertrophy (HYP). The CNN backbone extracts local morphological characteristics, while the Transformer module captures long-range dependencies, thereby producing more discriminative representations of complex biomedical ECG signals.

The proposed framework was extensively evaluated and achieved an overall classification accuracy of 93.60%, with high Precision, Recall, Specificity, F1-score, and AUC values. The confusion matrix, ROC analysis, comparative evaluation, and ablation study further demonstrated the effectiveness, robustness, and reliability of the proposed machine-learning framework. Overall, the integration of mRMR-based feature selection with CNN–Transformer learning provides a competitive approach for automated ECG-based cardiac disease diagnosis and biomedical signal analysis.

The framework will be further validated using additional public and clinical ECG datasets to investigate its generalization across diverse patient populations and acquisition conditions. Furthermore, lightweight CNN–Transformer architectures will be explored for real-time deployment in wearable and edge-based healthcare systems. Explainable artificial intelligence (XAI) and attention-visualization techniques will also be investigated to improve model interpretability, clinical decision support, and the practical applicability of machine-learning-based automated ECG diagnosis.

## Figures and Tables

**Table 1:** Summary of the patient-wise PTB-XL dataset partitioning strategy used in the proposed framework.

Dataset Partition	PTB-XL Folds	Purpose	Class Balancing	Feature Selection (mRMR)
Training set	Folds 1–8	Model training and parameter learning	Applied only to training data, if required	mRMR fitted using training data only
Validation set	Fold 9	Model selection and best-checkpoint selection	Not applied	Training-selected feature subset applied unchanged
Independent test set	Fold 10	Final unbiased performance evaluation	Not applied	Training-selected feature subset applied unchanged

**Table 2:** Class-wise mean and standard deviation of the temporal, statistical, spectral, and nonlinear ECG features extracted from the PTB-XL sub-epochs.

Feature	NORM (Mean ± SD)	MI (Mean ± SD)	STTC (Mean ± SD)	CD (Mean ± SD)	HYP (Mean ± SD)
Standard Deviation	0.1742 ± 0.0482	0.1901 ± 0.0633	0.1628 ± 0.0516	0.2103 ± 0.0604	0.1588 ± 0.0483
RMS Amplitude	0.1733 ± 0.0479	0.1891 ± 0.0630	0.1620 ± 0.0514	0.2092 ± 0.0601	0.1580 ± 0.0481
Peak-to-Peak	1.0112 ± 0.2312	1.0962 ± 0.2802	0.9829 ± 0.2504	1.1935 ± 0.2617	0.9645 ± 0.2325
Skewness	1.7309 ± 1.0534	1.3756 ± 1.2957	2.1914 ± 0.9408	0.1678 ± 1.7386	2.1719 ± 1.1802
Kurtosis	8.6600 ± 4.5480	8.4166 ± 4.7630	10.9607 ± 4.8669	7.9786 ± 3.7266	11.2918 ± 4.2982
Signal Energy	3.2332 ± 1.9460	3.9736 ± 3.0375	2.8862 ± 2.7354	4.7374 ± 2.8316	2.7285 ± 1.9698
Zero-Crossing Rate	0.1029 ± 0.0327	0.1143 ± 0.0487	0.1167 ± 0.0431	0.1075 ± 0.0382	0.1055 ± 0.0377
Dominant Frequency	3.9180 ± 2.4600	3.8380 ± 2.8404	4.7460 ± 2.5247	3.3260 ± 2.3329	4.4180 ± 2.4843
Spectral Centroid	7.3878 ± 1.6925	7.4410 ± 2.2918	7.8066 ± 1.8740	6.4031 ± 1.9425	7.6130 ± 1.7984
Spectral Bandwidth	4.9488 ± 1.3018	5.1522 ± 1.5469	4.6445 ± 1.3151	4.4631 ± 1.3336	4.6243 ± 1.3134
Spectral Entropy	0.6395 ± 0.0645	0.6280 ± 0.0816	0.6372 ± 0.0671	0.5912 ± 0.0739	0.6396 ± 0.0635
Permutation Entropy	0.7840 ± 0.0546	0.7982 ± 0.0650	0.7882 ± 0.0640	0.7633 ± 0.0650	0.7783 ± 0.0584
Hjorth Mobility	0.5220 ± 0.1022	0.5276 ± 0.1460	0.5333 ± 0.1180	0.4577 ± 0.1219	0.5226 ± 0.1119
Hjorth Complexity	1.5271 ± 0.2796	1.6501 ± 0.4985	1.4314 ± 0.2406	1.6421 ± 0.4436	1.4518 ± 0.2689

**Table 3:** Architecture and experimental configuration of the proposed CNN–Transformer classification model.

Parameter	Value
Input Representation	8 mRMR-selected ECG features
Input Shape	1 × 8
CNN Block 1	Conv1D, 64 filters, kernel size 3
CNN Block 2	Conv1D, 128 filters, kernel size 3
CNN Activation	ReLU
Normalization	Batch Normalization
Pooling	MaxPooling 1D
Transformer encoder block	2
Attention Heads	4
Embedding Dimensions	128
Feed-forward Dimension	256
Dropout	0.5
Total Trainable Parameters	298,885
Classifier	CNN Transformer
Optimizer	Adam
Loss Function	Categorical Cross-Entropy
Batch Size	32
Learning Rate	0.001
Epochs	45
Activation Function	ReLU
Output Layer	Softmax
Evaluation Metrics	Accuracy, Precision, Recall, F1-score, Specificity, ROC-AUC

**Table 4:** Quantitative performance evaluation of the proposed CNN–Transformer model on the PTB-XL dataset using Accuracy, Precision, Recall, Specificity, and F1-score.

Class	Precision	Recall	Sepecificity	F1-Score
NORM	0.9216	0.94	0.980	0.9307
MI	0.9394	0.93	0.985	0.9347
STTC	0.9388	0.92	0.985	0.9293
CD	0.9394	0.93	0.985	0.9347
HYP	0.9412	0.96	0.985	0.9505

**Table 5:** Comparison of the proposed CNN–Transformer framework with existing PTB-XL ECG classification methods.

Authors	Class	Data Points	Augmentation	Data Split	Evaluation Unit	Methods	Accuracy (%)
Kacprzak et al. [34]	5 superclasses: NORM, MI, STTC, CD, HYP	17,232 ECG recordings	Not explicitly reported	70% train/15% validation/15% test	ECG recording	CNN with Entropy Features	76.50
Bickmann et al. [35]	5 superclasses: NORM, MI, STTC, CD, HYP	21,801+ ECG recordings in PTB-XL dataset; model-specific subsets used	Not explicitly reported	PTB-XL stratified folds; fold 10 used for final evaluation	ECG recording	Feature-based vs. Time-Series ML Benchmark	83.90
Strodthoff et al. [18]	PTB-XL diagnostic tasks, including 5 diagnostic superclasses	21,837 ECG recordings	Not explicitly reported	Official folds 1–8 train, 9 validation, 10 test	ECG recording	ResNet/Inception Benchmark	86.40
Mehdi and Ali [38]	5 classes: NORM, MI, STTC, CD, HYP	21,834 ECG recordings before class balancing	Class balancing/oversampling applied	Folds 1–9 used for train/validation; fold 10 test	ECG recording	Simplified CNN–VAE	87.01
Elyamani et al. [15]	5 classes: NORM, MI, STTC, CD, HYP	21,799 ECG recordings	No	Folds 1–9 train; fold 10 test	ECG recording	Deep Residual 2D CNN	89.90
Proposed Method	5 mutually exclusive classes: NORM, MI, STTC, CD, HYP	21,837 original PTB-XL recordings; mutually exclusive five-class subset retained after filtering	No	Official patient-disjoint folds 1–8 train, 9 validation, 10 test	ECG-derived heartbeat/feature representation	mRMR + CNN–Transformer	93.60

**Table 6:** Ablation study of the proposed CNN–Transformer framework on the PTB-XL dataset.

Model Configuration	VMD	Temporal	Statistical	CNN	Transformer	mRMR Feature Selection	Accuracy (%)
CNN Baseline	✓	✓	✓	✓	✗	✗	88.41
CNN–Transformer	✓	✓	✓	✓	✓	✗	91.82
CNN + mRMR	✓	✓	✓	✓	✗	✓	90.74
Without VMD	✗	✓	✓	✓	✓	✓	87.69
Without Temporal	✓	✗	✓	✓	✓	✓	90.34
Without Statistical	✓	✓	✗	✓	✓	✓	92.62
Proposed CNN–Transformer + mRMR	✓	✓	✓	✓	✓	✓	93.60

✓ (included) ✗ (Not Included).

## Data Availability

The dataset used in this study is publicly available. The PTB-XL electrocardiography (ECG) dataset can be accessed through the official PhysioNet repository at PhysioNet PTB-XL Dataset https://physionet.org/content/ptb-xl/1.0.1/ (accessed on 27 July 2026). All experiments in this study were conducted using the publicly available PTB-XL dataset without any modification to the original annotations.
